# Antimicrobial Peptides and Proteins: From Nature’s Reservoir to the Laboratory and Beyond

**DOI:** 10.3389/fchem.2021.691532

**Published:** 2021-06-18

**Authors:** Tanumoy Sarkar, Monikha Chetia, Sunanda Chatterjee

**Affiliations:** Department of Chemistry, Indian Institute of Technology, Guwahati, India

**Keywords:** antimicrobial peptides, antimicrobial resistance, natural and synthetic AMPs, antimicrobial activity, cytotoxicity, protease resistance, AMP delivery strategies

## Abstract

Rapid rise of antimicrobial resistance against conventional antimicrobials, resurgence of multidrug resistant microbes and the slowdown in the development of new classes of antimicrobials, necessitates the urgent development of alternate classes of therapeutic molecules. Antimicrobial peptides (AMPs) are small proteins present in different lifeforms in nature that provide defense against microbial infections. They have been effective components of the host defense system for a very long time. The fact that the development of resistance by the microbes against the AMPs is relatively slower or delayed compared to that against the conventional antibiotics, makes them prospective alternative therapeutics of the future. Several thousands of AMPs have been isolated from various natural sources like microorganisms, plants, insects, crustaceans, animals, humans, etc. to date. However, only a few of them have been translated commercially to the market so far. This is because of some inherent drawbacks of the naturally obtained AMPs like 1) short half-life owing to the susceptibility to protease degradation, 2) inactivity at physiological salt concentrations, 3) cytotoxicity to host cells, 4) lack of appropriate strategies for sustained and targeted delivery of the AMPs. This has led to a surge of interest in the development of synthetic AMPs which would retain or improve the antimicrobial potency along with circumventing the disadvantages of the natural analogs. The development of synthetic AMPs is inspired by natural designs and sequences and strengthened by the fusion with various synthetic elements. Generation of the synthetic designs are based on various strategies like sequence truncation, mutation, cyclization and introduction of unnatural amino acids and synthons. In this review, we have described some of the AMPs isolated from the vast repertoire of natural sources, and subsequently described the various synthetic designs that have been developed based on the templates of natural AMPs or from *de novo* design to make commercially viable therapeutics of the future. This review entails the journey of the AMPs from their natural sources to the laboratory.

## Introduction

In the wake of the post-pandemic resurgence, the greatest challenge faced by the civilized world are the antimicrobial infections. Rapidly growing antibiotic resistance (Mathur and Singh, 2005) accompanied by the slowdown of the development of the newer antibiotics (Ventola, 2015) in the recent past, is a reason for major concern. Scientists have been trying to look for alternate strategies or new classes of molecules to combat against the microbes. Antimicrobial peptides (AMPs) are small proteins present in a various plant and animal species that act as the first line of defense against the microbes. Their existence in nature for over millions of years and sustained capability to combat antimicrobial infections is a testimony of complete absence of or very slow development of resistance against them (Yu et al., 2018). This makes them specifically desirable in comparison to the antibiotics, which develop resistance pretty fast. The absence/slow development of resistance against microbes may be attributed to the presence of various modes/mechanisms of action of the AMPs against the bacteria in comparison to the fixed targets used by the antibiotics (Papo and Shai, 2003; Brogden, 2005; Nicolas, 2009). Additionally, AMPs breakdown into amino acids unlike other therapeutics, which might generate potentially harmful metabolites.

**FIGURE 1:**
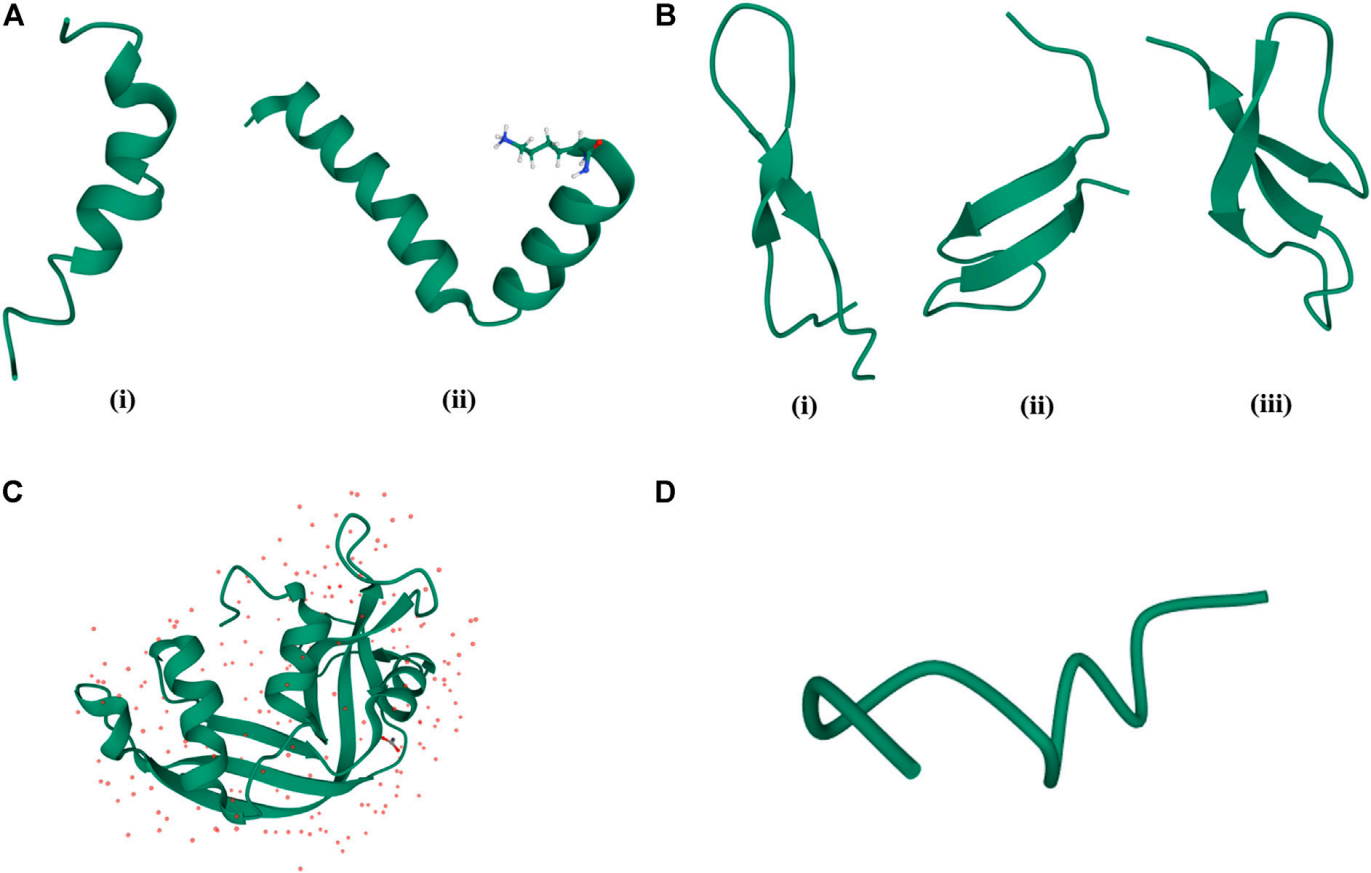
Classification of AMPs based on their structures. **(A)** α-helical AMPs: (i) Magainin (PDB code—2LSA), (ii) Cecropin (PDB code—2LA2). **(B)** β-sheet AMPs: (i) Bovine Lactoferrin (PDB code—1LFC), (ii) Hepcidin (PDB code—2KEF), (iii) Rabbit Kidney Defensins (PDB code—1EWS). **(C)** α-helix, β-sheet mixed AMPs: RNase 2 (PDB code—1GQV). **(D)** Random coil structure of AMPs: Cys deleted protein (PDB code—2MQ5).

AMPs are produced as secondary metabolites and contain upto about 50 amino acid residues. They greatly vary in their sequence, secondary structural preferences, and in their modes of action. While a large variety of AMPs are cationic in nature (CAMPs) containing about +2 to +9 charge in them (Cascales et al., 2018), there can be anionic AMPs (AAMPs) (Harris et al., 2009; Lai et al., 2002; Steffen et al., 2006) and completely hydrophobic AMPs (Epand and Vogel, 1999) as well. Secondary structures of AMPs vary from α-helices [magainin (Zasloff, 1987)], protegrin (Fahrner et al., 1996), LL-37 (Scott et al., 2002), β-sheets [α, β-defensins (Selsted et al., 1985a; Selsted et al., 1985b)], drosomycin (Fehlbaum et al., 1994), thionins (Stec, 2006), plectasin (Mygind et al., 2005), mixed αβ structures [RNase 2, RNase 3 (Hamann et al., 1990)] to extended helices and loop structures (Hancock, 1997; Jenssen et al., 2006) (Figure 1). While membranolysis (Sitaram and Nagaraj, 1999) or disruption in the synthesis of the cell membrane/cell wall is considered as one of the major modes of action of the AMPs, other AMPs have intracellular targets and interfere with the vital intracellular processes (Scocchi et al., 2016). Some AMPs are known to have an immunomodulatory effect on the innate immune system of the host (Hancock and Sahl, 2006). AMPs can belong to various chemical classes like cationic (Findlay et al., 2010), hydrophobic (Schmidtche et al., 2014), amphipathic, disulfide bonded (Krause et al., 2000), cyclic (Dartois et al., 2005) and lipidated (Hu et al., 2012).

**GRAPHICAL ABSTRACT:**
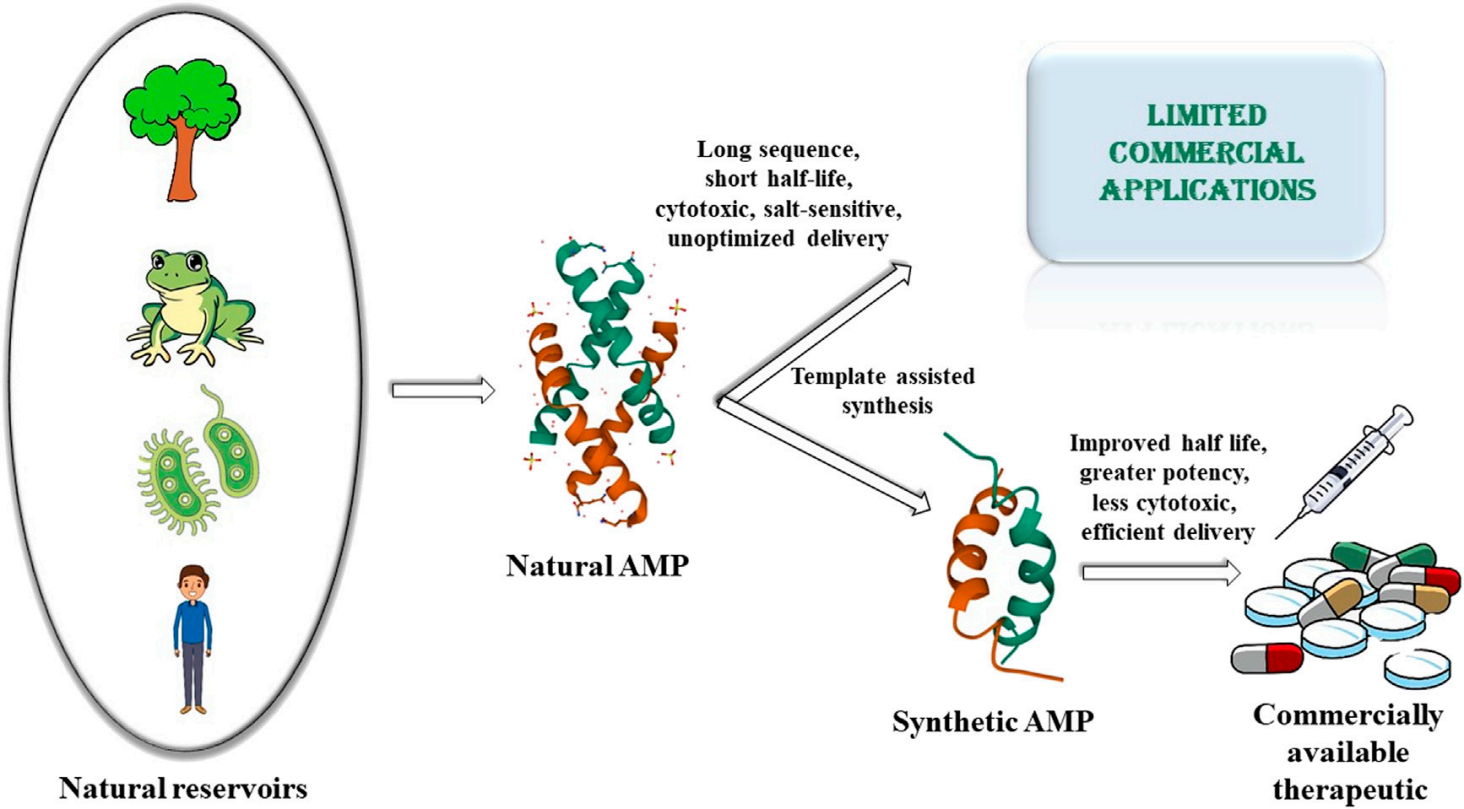


Over the last couple of decades, thousands of AMPs have been isolated from different kingdoms of life (Mishra et al., 2018). There are several databases like APD3 (Wang et al., 2016), CAMP (Waghu and Thomas, 2020), DrAMP2.0 (Kang et al., 2019) etc. which catalog the various AMPs that are known to date. AMPs are produced by microorganisms to protect them from other microbes. Some of the AMPs are shared by different classes of eukaryotes like plants and animals (defensins and cyclotides), others are only specific to plants (heveins), while others are specific to certain plant families (snakins). When compared to the other organisms, plants tend to have a higher amount of AMPs due to gene duplications or polyploidy. This may also be attributed to their immobility, which disables them to avoid the biotic and environmental conditions. Thionins, plant defensins, heveins, snakins are some plant-derived AMPs (Nawrot et al., 2014; Tam et al., 2015). Several AMPs like japonicin 2, nigrocin 2, temporin, dermaseptin, magainin, buforin II (Conlon and Mechkarska, 2014; Patocka et al., 2019; Casciaro et al., 2020) have been isolated from the amphibians (Wang et al., 2016), which have broad-spectrum antimicrobial activities against bacteria, fungi, yeast, protozoa and virus along with some potential in cancer therapy (Rabanal and Cajal, 2016). Cepropin, thanatin, defensin, drosomycin, apidaecin, abaecin, melittin are some of the insect derived AMPs (Mylonakis et al., 2016). Crustaceans produce several AMPs like callinectin, homarin, penaeidin, hyastatin, arasin (Zanjani et al., 2018). Mammals produce AMPs like defensins, histatins, LL-37, indolicidin, protegrins and lactoferrins (Wang, 2014; Dutta and Das, 2016). In animals, AMPs are isolated mostly from tissues and organs that are exposed to airborne pathogens like skin, eyes, ears, mouth, airways, lungs, intestines, urinary tract etc. and acts as the first line of innate immune defense (Schauber and Gallo, 2008; Hilchie et al., 2013).

In spite of being a very promising alternative class of therapeutics, only a very few of the AMPs have been commercialized so far. This is owing to several inherent disadvantages of natural AMPs. These include limited quantities of AMPs isolated from the natural sources, uneconomic synthesis of longer AMP sequences, folding hurdles related to the natural AMPs, short half-life of the natural AMPs owing to protease degradability systemic toxicity and delivery issues to the target site. Inspired by nature and to address the shortcomings of the natural AMPs, research on synthetic AMPs has been explored extensively over the last few decades. In this review, we briefly recounted the journey of the natural AMPs from different kingdoms of life to the mainstream of synthetic research. As the field of AMP research is huge, and there are several elaborate reviews on the subtopics discussed here, this review does not claim to be exhaustive in nature. Instead, we have tried to represent and discuss 1) the various chemical classes of AMPs from different kingdoms of life which act as their natural reservoirs, 2) major shortcomings of the natural AMPs which limit their commercial applications, 3) how natural AMPs have influenced and inspired the synthetic AMP research and 4) how the synthetic AMP research addresses the shortcomings of the natural AMPs to make effective therapeutic antimicrobials of the future. In short, this review very briefly depicts the current scenario in the pursuit of the development of AMPs as future commercial therapeutic agents.

## Natural Antimicrobial Peptides

There are different ways in which AMPs can be classified. These rely on their physicochemical properties, secondary structure, sources, modes of action etc. In this review, we have broadly classified the AMPs into 1) cationic natural AMPs (Table 1), 2) Cyclic AMPs (Table 1) and 3) AMPs rich in specific amino acid residues like tryptophan (Trp), proline (Pro), histidine (His) and glycine (Gly) (Table 1).

**TABLE 1:** Natural antimicrobial peptides (aa = amino acid; res = residues).

Name of the AMPs	Natural source	Physicochemical properties	Structure	Activity	References
Natural cationic antimicrobial peptides					
α Defensins	Mammals including humans, monkeys; several rodent species; plants	Cationic; three disulfide bridges; Mol wt.: 3–4 kDa	Triple-stranded β- sheet	Antiviral, virucidal, antifungal	Ganz (2001); Lehrer (2004)
				Anticancer activity (HNP1,2,3), chemotactic activity	
β Defensins	Almost all mammals, insects, plants	Cationic; three disulfide bridges; Mol wt.: 2–6 kDa	β- sheet	Virucidal, anticancer activity (hBD1), chemotactic activity	Diamond et al. (2009)
Cathelicidin LL-37	Humans	37 aa res; Cationic Sequence: LLGDFFRKSKEKIGKEFKRIVQRIKDFLRNLVPRTES	α-helical Three parts: N- and C-terminal α-helices (2–30 aa res.), unstructured C-terminal tail (31–37 aa res)	Virucidal, antifungal antiparasitic activity, anticancer activity, chemotactic activity, apoptosis, wound healing	Gudmundsson (1996); Yamasaki et al. (2006); Braff and Gallo (2006)
Hepcidin 25 (LEAP-1)	Humans	Four disulfide bridges; Sequence - DTHFPICIFCCGCCHRSKCGMCCKT (Disulfide bridges: 7–23, 10–13, 11–19, 14–22)	β- sheet	Antifungal activity, active against gram-positive and gram-negative bacteria	Krause et al. (2000)
RNase 2 (EDN)	Humans (in large specific granules of human eosinophilic leukocytes)	Cationic; also called eosinophil derived neurotoxin (EDN); Non-secretory ribonuclease; Mol. Wt. ∼ 18 kDa	α,β-mixed structure	Antiviral activity, antiparasitic activity	Hamann et al. (1990); Domachowske et al. (1998)
RNase 3 (ECP)	Humans (in the eosinophil primary matrix)	Cationic; also known as eosinophil cationic protein (ECP) Mol. Wt.: 18–22 kDa	α,β-mixed structure	Antiviral activity, Antiparasitic activity	Hamann et al. (1990)
Lysozyme	Found in most mammals including humans; Bacteria, funci	Positively charged enzymes; aa res > 100	Structure comprising of large number of α-helices, β-sheet and random coils	Catalyzes the hydrolysis of 1,4-β-linkages between N-acetylmuramic acid and N-acetyl-D-glucosamine residues in peptidoglycan, which is the major component of gram-positive bacterial cell wall	Fleming (1922)
Thionins	Plants	Contains 45–47 amino acids and are usually high in cyst, lysine, and arginine; highly charged and basic; Mol. Wt.: ∼5 kDa	α,β-mixed structure	Bacteria, Fungi, yeast	Bohlman and Apel (1991)
Cecropins	Insects; first isolated from the hemolymph of Hyalophora cecropia. Various homologues also found in mammals	31–39 aa res; Cationic Cecropin A: KWKLFKKIEKVGQNIRDGIIKAGPAVAVVGQATQIAK; Cecropin B: KWKVFKKIEKMGRNIRNGIVKAGPAIAVLGEAKAL; Cecropin P1: SWLSKTAKKLENSAKKRISEGIAIAIQGGPR	α-helical	Broad-spectrum activity against gram-positive and gram-negative bacteria, fungi	Steiner et al. (1981)
Mellitin	Constitutes 40–60% of dry honeybee (Apis mellifera) venom	26 aa res; Cationic; Sequence; GIGAVLKVLTTGLPALISWIKRKRQQ-NH_2_	α-helical in the presence of membrane mimics	Acts by inserting itself across the phospholipid bilayer, and interact between biomembrane and proteins; Antibacterial, anti-inflammatory; Effective against MRSA	Habermann (1972)
Jelleines	Isolated from the western honey bee or European honey bee (Apis mellifera)	8–9 aa res; Sequences: Jelleine-I (PFKLSLHL-NH_2_), jelleine-II (TPFKLSLHL-NH_2_), jelleine-III (EPFKLSLHL-NH_2_) and jelleine-IV (TPFKLSLH-NH_2_)	-	Active against yeast, fungi, gram-positive and gram-negative bacteria	Fontana et al. (2004)
Esculatin 1a	Skin of frogs (*Rana esculenta*)	Sequence–H_2_N-GIFSKLAGKKIKNLLISGLKNVGKEVGMDVVRTGIDIAGCKIKGEC-COOH	Disordered in solution but adopts an α-helical structure when associated with the membranes mimics	Broad-spectrum antimicrobial activities against gram-positive and gram-negative bacteria	Simmaco et al. (1994)
Esculatin 1b	Skin of frogs (*Rana esculenta*)	Sequence–H_2_N-GIFSKLAGKKLKNLLISGLKNVGKEVGMDVVRTGIDIAGCKIKGEC-COOH	Disordered in solution but adopts an α-helical structure when associated with the membranes mimics	Broad-spectrum antimicrobial activities against gram-positive and gram-negative bacteria	Simmaco et al. (1994)
Esculatin-2CHa	Skin of frogs (*Rana chiricahuensis*)	37 aa res; Cationic Sequence: GFSSIFRGVAKFASKGLGK DLAKLGVDLVACKISKQC	α-helical	Broad-spectrum antimicrobial activities, Type 2 Diabetes	Attoub et al. (2012)
Temporins	Isolated from the skin secretion of European red frog (*Rana temporaria*); Also secreted by temporal glands of elephants	10–14 aa res; Cationic Temporin A: FLPLIGRVLSGIL-NH_2_; Temporin B: LLPIVGNLLKSLL-NH_2_; Temporin C: LLPILGNLLNGLL-NH_2_; Temporin D: VLPIIGNLLNSLL-NH_2_; Temporin E: VLPIIGNLLNSLL-NH_2_; Temporin F: FLPLIGKVLSGIL-NH_2_; Temporin G: FFPVIGRILNGIL-NH_2_ Temporin H: LSP---NLLKSLL-NH_2_: Temporin K: LLP---NLLKSLL-NH_2_; Temporin L: FVQWESKFLGRIL-NH_2_	α-helical	Gram-positive bacteria, MRSA, vancomycin resistant enterococcus faecium, enterococcus faecalis	Simmaco et al. (1996)
Nigrocin 1 and 2	Isolated from the Korean frog (*Rana nigromaculata*)	Cationic; Gly rich; one disulfide bridge	Amphipathic α-helix in microbial membrane mimics	Broad-spectrum antimicrobial, gram-positive and gram-negative bacteria, protozoa and fungi	Park et al. (2001a)
Buforin II	Isolated from the stomach tissue of Asian toad (*Bufo bufo gargarizans*)	21 aa res; Pro hinge important for activity	α-helical	Broad-spectrum antimicrobial, non-membranolytic, affinity for DNA, RNA	Park et al. (1996)
Natural cyclic peptides					
Syringomycins	*Pseudomonas syringae pv. syringae*	Cyclic lipopeptides; Lipodepsinona-peptide comprising of a closed ring of nine non-ribosomally synthesized aa res bonded to a fatty-acid hydrocarbon tail	Combination of a lipid and a peptide. Peptide portion containing amino acids in a cycle. Some of the amino acids are rare, e.g. in Syringomycin-E: 2,4-diaminobutyric acid and β-hydroxy aspartic acid	Active against gram-positive bacteria, mycelia	Fukuchi et al. (1992)
Massetolide A	Pseudomonas fluorescence SS101	Cyclic lipopeptide; consists of a 9-amino-acid cyclic oligopeptide linked to 3-hydroxydecanoic acid	Consists of a 9-amino-acid cyclic oligopeptide linked to 3-hydroxydecanoic acid	Affects plant pathogenic oomecytes	Tran et al. (2007)
Amphisin	Pseudomonas sp. strain DSS73	Cyclic lipoundeca-peptide; Amphiphilic having biosurfactant properties; Sequence: β-hydroxydecanoyl-_D_L-_D_D-_D_-allo-T-_D_L-_D_L-_D_S-_L_L-_D_-Q-_L_L-_L_I-_L_D.	The peptide is a lactone, linking Thr3 Oγ to the C-terminal.Helical structure (310-helix)	Antagonistic against oomycete *Pythium ultimum*	Andersen et al. (2003)
Lokisin	Pseudomonas sp. strain DSS41	Cyclic lipoundeca-peptide; Leu rich having a D/L leu ratio of 3:2 Sequence: β-hydroxydecanoyl -_D_L-_D_D-_D_-allo-T-_D_L-_D_L-_D_S-_L_L-_D_S-_L_L-_L_I-_L_D.	The peptide is a lactone, linking Thr3 Oγ to the C-terminal-	Antagonistic against oomycete *Pythium ultimum*	Sørensen and Nielsen (2002)
Tensin	*Pseudomonas Fuorescens* strain 96.578	Cyclo Lipopeptide: Amphiphilic having biosurfactant properties; Sequence: β-hydroxydecanoyl-DL-DD-D-*allo*-T-DL-DL-DS-_L_L-DQ-_L_L-_L_I-_L_Q.	The peptide is a lactone linking the Thr3 Oγ atom to the C-terminal C atom. Helical structure (310-helix)	Antagonistic against *Rhizoctonia solani*	Nielsen et al. (2000)
Viscosinamide	*Pseudomonas fluorescens* DR54	Cyclo Lipopeptide; Depsipeptide having biosurfactant properties	Cyclized via an ester bond between the C-terminal carbonyl and the side chain of a D-allo-Thr at position 3, leading to a 7-residue macrocycle	Antagonistic against oomycete *Pythium ultimum* and *Rhizoctonia solani*	Nielsen et al. (1999)
Iturins	*Bacillus*	Cyclic lipopeptide Nonribosomal lipopeptide family of seven residues of α and β aa res	Peptide ring of seven aa res including an invariable D-Tyr2, with the constant chiral sequence LDDLLDL closed by a C14-C17 aliphatic β aa	Exhibit strong antifungal activities against a wide variety of pathogenic yeasts and fungi; Shows selective antibacterial activity	Maget-Dana and Peypoux (1994)
Fengycins	*Bacillus subtilis*	Cyclic lipopeptides 10-aa res; β-hydroxy fatty acid	The structure is com-posed of a β-hydroxy fatty acid linked to a peptide part comprising 10 aa, where 8 of them are organized in a cyclic structure	Prevents growth of mycelia of *Fusarium oxysporum*, *R. solani* and *B. cinerea*	Vanittanakom et al. (1986)
Surfactins	*Bacillus subtlis*	Cyclic lipopeptides; 7 aa res; β-hydroxy fatty acid of variable length; Amphipathic; Consists of a peptide loop of seven amino acids (_L_E-_L_L- _D_L,-_L_V,-_L_D-_D_L-_L_L) and a β-hydroxy fatty acid of variable length, thirteen to fifteen carbon atoms long	‘Horse saddle’ conformation in solution	Antimicrobial against *Candida albicans*, *Lactococcus lacti* subsp. Lactis, *Mycobacterium smegmatis*, *Staphylococcus aureus*	Arima et al. (1968)
Balliomycin D	*Bacillus subtlis*	7 aa res; β-amino fatty acids with 14–16 C atoms	The lipid moiety consists of 3-amino-12-methyltridecanoic acid or 3-amino-12-methyltetradecanoic acid; the peptide moiety contains one residue of each of the following seven amino acid: DN-LD-LE-LP-DS-LT-DY	Inhibits *B. cinerea*	Peypoux et al. (1981)
Mycosubtilin	*Bacillus subtlis*	Iturin derivative; 7 aa res; β-amino fatty acids with 14–16 C atoms	Mycosubtilin is a heptapeptide, cyclized in a ring with a β-amino fatty acid. The peptide sequence is composed of LN-DY-DN-LQ-LP-DS-LD	Strong antifungal activity	Walton and Woodruff (1949)
Fusaridins	*Bacillus Polymyxa*	Cyclic lipopeptides Group of depsipeptides with an unusual fatty acid, 15-guanidino-3-hydroxypentadecanoic acid moiety bound to a free amino group	-	Controls Phytopthora blight infection in red pepper caused by *Phytopthora capsica*	Kajimura and Kaneda (1997)
Gramicidin S	*Bacillus brevis*	Cyclicdeca-peptide; Composed of five different amino acids, each one used twice within the ring structure. Sequence: cyclo-(^D^Phe-Pro-Val-Orn-Leu)_2_	C2-symmetric β-sheet structure that is stabilized by four interstrand hydrogen bonds between the Leu and Val residues	Antifungal activity and sporicidal activity	Gause and Brazhnikova (1994)
Valinomycin	*Streptomyces* species (*S. fulvissimus*)	Cyclopeptide Cyclic dodeca-depsipeptide; Neutral ionophore	Made of twelve alternating D- and L-aas and esters: D- and L-Val, D-α-hydroxyisovaleric acid, and L-lactic acid to form a macrocyclic molecule	Insecticidal, acaricidal, antifungal antioomecyte activity. Modulates the transport of potassium ions across biological membranes	Brockmannan Scttmidt-Kastner (1955)
Polyoxins	*Streptomyces cacaoi*	Cyclopeptide; nucleoside-peptide antibiotics	Polyoxin are pyrimidine nucleosides linked to a peptide moiety	Competitive inhibitors of chitin synthase	Isono and Suzuki (1979)
Arthrichitin	*Arthinium phaeospermum*	Cyclic depsipeptide; Consists of serine, β-keto tryptophan, glutamic acid, and 2,4-dimethyl-3-hydroxydodecanoic acid units Sequence: Cyclo (3-hydroxy-2,4-dimethyldodecanoyl-L-alpha-glutamyl-beta- oxotryptophyl-L-seryl)	-	Inhibits chitin biosynthesis/anti-fungal	Vijayakumar et al. (1996)
Neopeptins	*Streptomyces* sp	Cyclic lipopeptides; Nonadepsi-peptide; 9 unnatural aa res and an acyl chain	Cyclic; unusual structure and a varying lipid tail	Antifungal. Completely inhibits *Glomerella cingulata, P. oryzae*, *Cochliobolus miyabeanus*, and *Colletotrichum lagenaium*	Satomi et al. (1982)
Tyrocidins	Soil bacteria *Bacillus Brevis*	Cyclic decapeptides rich in Phe; Sequence: Tyrocidine A: cyclo (_L_N-_L_Q-_L_W-_L_V-_L_Orn-_L_L-_D_F-_L_P-_L_F-_D_F). Tyrocidines B–D are isoforms of tyrocidine A with specific replacements of one (Tyrocidine B, L-Phe→ L-Trp at position 3), two (Tyrocidine C, L–Trp→L–Phe and D–Trp→D–Phe at positions 3 and 4), or three amino acid residues (Tyrocidine D, the changes observed in tyrocidine C in addition to L–Trp→L–Tyr at position 7)	Cyclic, Homodectic	Broad-spectrum anti-fungal activity	Roskoski et al. (1970)
Aureobasidins	*Aureobasidins pullulans*	Cyclic depsipeptide; 8 lipophilic aa res, 1 α-hydroxy acid	Cyclic depsipeptide consisting of eight α aa units and one hydroxy acid unit. Sequence: 2(R)-hydroxy-3(R)-methylpentanoic acid, β-hydroxy-N-methyl-LV, N-methyl-LV-LP-allo-LI-N-methyl-LF-LL-LF.	Antifungal and antiprotozoal	Takesako et al. (1991)
Cyclotides	Plant	28–37 aa res; head to tail cyclization; three disulfide bridges	Having a head-to-tail cyclic backbone and three conserved disulfide bonds that form a CCK motif	Anti-HIV, incesticidal, antimicrobial	Craik et al. (2004)
Kalata B8	Oldenlandia affinis	31 aa res; Sequence: GSVLNCGETCLLGTCYTTGCTCNKYRVCTKD	Kalata B8 contains the CCK motif characteristic of cyclotides but has some interesting differences, including disordered loops 1 and 6 (of a total of 6 loops)	Uterotonic, anti-HIV, antimicrobial, insecticidal, antihelmintic, and molluscidal properties	Daly et. al. (2006)
Hevein	*Hevea brasiliensis* (rubber tree)	Cys rich; 4/5/3 disulfide bridges; Gly rich	The structure is folded into a series of loops all linked together by four disulfide bonds	Antimicrobial, antifungal activities	Van Parijs et al. (1991)
Daptomycin	Streptomyces roseosporus	13 aa res; cyclic lipopeptide; Contains two non-proteinogenic amino acids: L-kynurenine (Kyn) and L-3-methylglutamic acid (mGlu)	Consists of 13 amino acids, 10 of which are arranged in a cyclic fashion	Gram-positive bacteria	Steenbergen et al. (2005)
Natural antimicrobial peptides rich in W, P, H, G					
Histatin 1	Human saliva	38 aa res; Sequence: DSHEKRHHGYRRKFHEKHHSHREFPFYGDYGSNYLYDN	-	Antifungal activity against *C. albicans*	Oppenheim et al. (1988)
Histatin 3	Human saliva	32 aa res; Sequence: DSHAKRHHGYKRKFHEKHHSHRGYRSNYLYDN	-	Antifungal activity against *C. albicans*	Oppenheim et al. (1988)
Histatin 5	Human submandibular and parotid glands	24 aa res; Cationic; His rich; Sequence: DSHAKRHHGYKRKFHEKHHSHRGY	α-helical in non-aqueous solvents	Virucidal, antifungal against *C. albicans*	Oppenheim et al. (1988)
KDAMP	Humans	19 aa res; Gly rich; Sequence: RAIGGGLSSVGGGSSTIKY	Coil structure with low α-helical content	Active aginast gram-negative bacteria, e.g. *P. aeruginosa*	Tam et al. (2012)
Human lactoferricin (LfcinH)	Humans	49 aa res; Cationic; Trp rich	Shows a helical content from Gln14 to Lys29 in the membrane mimetic solvent but a nonexistent β-sheet character in either the N- or C-terminal regions of the peptide	Antifungal	Hunter et al. (2005)
Bovine lactoferricin (LfcinB)	Bovine	25 aa res; cationic; Trp rich	Distorted antiparallel β sheet	Inhibits the growth of a large variety of bacteria, fungi, viruses, parasites	Hwang et al. (1998)
Puroindolins	Plants, wheat grain	Cationic; Trp rich	α-Helical	Antibacterial and antifungal	Blochet et al., 1991
Lebocins	Silkworm *Bombyx mori*	32 aa res; Pro rich; O-glycosylated; Sequence of lebocin 1: DLRFLYPRGKLPVPTPPPFNPKPIYIDMGNRY-NH2. The primary sequence of lebocins 1 and 2 differ only in their sugar moiety. Lebocin 3 has the same sequence as lebocin 2, except that aa res 16 is Leu instead of Pro	-	Active against gram-negative and gram-positive bacteria, antifungal	Hara and Yamakawa (1995)
Drosocin	*Drosophila melanogaster*	19 aa res; Pro rich; O-glycosylated; Sequence: GKPRPYSPRPTSHPRPIRV-NH_2_	β-turns	Binds bacterial DnaK, ribosomes	Bulet et al. (1993)
Pyrrhocoricin	*Pyrrhocoris apterus*	20 aa res; cyclic; Trp rich; Sequence: VDKGSYLPRPTPPRPIYNRN-NH2	α,β-mixed structure	Binds bacterial DnaK, transduces cell membrane of parasite cryptosporidium parvum	Cociancich et al. (1994)
Metchnikowin	*Drosophila melanogaster*	26 aa res; Trp rich	α,β-mixed structure	Gram-positive bacteria, antifungal	Levashina et al. (1995)
Calcitermin C	Human airways	15 aa res; Sequence: VAIALKAAHYHTHKE	α-helical	Gram-negative bacteria	Cole et al. (2001)
Attacins	*Hyalophora cecropia*	Attacins A-D:190 aa res, Gly rich, basic; Attacins E-F: 190 aa res, Glycine rich, acidic	Attacins adopt a random coil secondary structure in an aqueous buffer	Gram-negative bacteria	Hultmark et al. (1983)
Diptericins	Insects; first isolated from the blowfly *Phormia terranova*	Gly rich; Mol wt.: 8–9 kDa	High β-turn content	Gram-negative bacteria	Dimarcq et al. (1988)
Persulcatusin	*Ixodes persulcatus*	Cationic; Gly rich; Three S-S bonds Sequence: GFGCPFNQGACHRHCRSIGRRGGYCAGLFKQTCTCYSR-NH_2_	α,β-mixed structure	MSSA and (MRSA)	Saito et al. (2009)

### Cationic Antimicrobial Peptides

**FIGURE 2:**
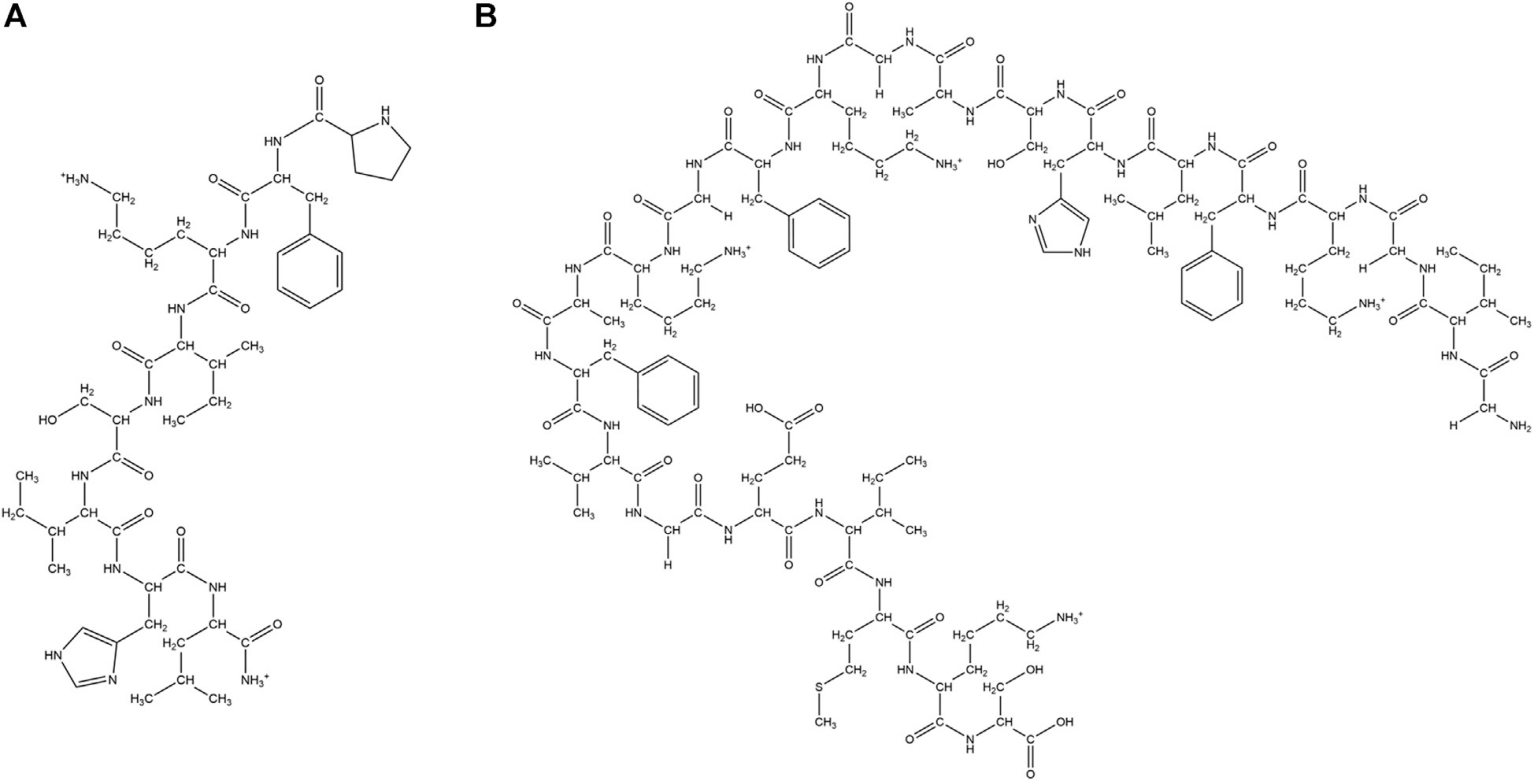
Examples of natural cationic AMPs. **(A)** Jelleine −1. **(B)** Magainin.

A large class of natural AMPs have positive charge (+2 to +9) due to the abundance of Lysine (Lys) and Arginine (Arg) residues (Hancock, 1997; Hancock and Rozek, 2002; Powers and Hancock, 2003; Brown and Hancock, 2006; Fernández-Vidal et al., 2007; Mangoni et al., 2007; Mookherjee et al., 2020) in them (Figure 2 and Table 1). This gives an electrostatic advantage to the AMPs in binding to the negatively charged microbial membranes. Often these peptides have amphipathic structures where the positive part of the AMP is separated from the hydrophobic part of the AMP in the tertiary structure. Most of the cationic AMPs have membrane disruptive/lytic mode of action.

Thionins are a class of small cationic plant AMPs rich in Lys, Arg and Cysteine (Cys) residues and are present in all the crucial plant tissues from endosperms to leaves. They are toxic against bacteria, fungi and yeasts. To date about 100 individual thionins have been isolated from about 15 plant species (Stec, 2006; Hussain et al., 2014; Kul'ko et al., 2016). Thionins have two antiparallel α helices and an antiparallel double stranded β-sheet with three or four disulfide bridges. Puroindolins (PINs) are small basic proteins isolated from wheat endosperms that have a unique Trp rich domain and five disulfide bridges. PINs have a conserved tertiary structure that is formed from four helices connected by variable loops, the tertiary structure being held together by five disulfide bridges. Antimicrobial activity of PINs is related to the interactions with the microbial membranes. Defensins are another class of small, cationic AMPs which are rich in Arg and Cys residues. They are present in various domains of life like plants, insects, animals and humans, and are highly active against a large range of microorganisms. Defensins have β-hairpin structures stabilized by three/four disulfide bonds. Defensins bind to membranes to form pore like membrane defects that lead to efflux of intracellular materials and eventual cell death. Plant defensins have antibacterial (Sitaram, 2006), antifungal (Gao et al., 2000), α-amylase and trypsin inhibitory activities (Wijaya et al., 2000). They are also active against yeast, oomycetes and necrotrophic pathogens (Segura et al., 1998; Van der Weerden et al., 2010). Additionally, they manifest anticancer and antiviral activities (Ngai and Ng, 2005; Wong and Ng, 2005). Plant defensins have a conserved structure with three stranded β-sheet and a helix stabilized by four disulfide bridges. Human defensins are of three types-α, β and θ. There are six subclasses of α defensins containing three conserved disulfide connectivities in between Cys ^I−VI^, Cys ^II−IV^ and Cys ^III−V^ (Ganz, 2001). β-defensins are longer in sequence than the α defensins and have Cys^I−V^, Cys^II−IV^, Cys^III−VI^ disulfide bond connectivities. There are various subclasses of human β-defensins (hBD). While hBD1 and hBD2 are rendered inactive in the presence of physiological salt concentrations (Goldman et al., 1997; Bals et al., 1998), hBD3 retains its activity against *Staphylococcus aureus* (*S. aureus*) and vancomycin resistant *Enterococcus faecium* at physiological salt concentrations (Harder and Bartels, 2001). Bioinformatics approaches have led to the identification of 23 additional human β-defensins from the disulfide bond connectivities, many of which have antimicrobial activities. Insect defensins are about 29–34 amino acids long with activity against both gram-positive and gram-negative bacteria (Hoffmann and Hetru, 1992), though the virulence against the gram-positive bacteria (*S. aureus*) (Hetru et al., 2003) is more than the gram-negative family (Gomes and Fernandes, 2010).

Cecropins are a family (seven subclasses) of small, cationic AMPs isolated from the hemolymph of giant silk moth *Hyalophora cecropia* (Steiner et al., 1981). Cecropins are active against gram-positive, gram-negative bacteria and fungi. Cecropins form pores in the bacterial membranes, cause membrane depolarization and lysis (Moore et al., 1996; Bechinger and Lohner, 2006). Bactericidin, lepidopteran and sarcotoxins are structurally related to cecropins (Ouyang et al., 2015). Melittin, a 26 amino acid residue long positively charged peptide hormone present in bee venom, is effective against several strains of bacteria (Jamasbi et al., 2018; Chen et al., 2016). Melittin is also reported to have anti-inflammatory properties (Lee and Bae, 2016) and activity against *xanthomonas oryzae*, a pathogenic bacteria causing destructive bacterial disease in rice (Shi et al., 2016). Melittin interacts with negative microbial membranes, leads to pore formation, efflux of intracellular material and eventual cell death (Rady et al., 2017). Melittin forms an amphipathic helical structure in the presence of microbial membrane mimics. The activity of melittin is highly dependent on a Pro residue in its primary sequence (Jamasbi et al., 2016). Melittin causes apoptosis in *C. albicans* (*Candida Albicans*) through reactive oxygen species (ROS) mediated mitochondria and caspase pathway (Lee and Lee, 2014). Melittin exhibits strong synergistic effects with the conventional antibiotics against MDR isolates of *Acenatibactor baumanni* and *Pseudomonas aeruginosa* (*P. aeruginosa*) (Akbari et al., 2019) and effective antibacterial activity against Methicillin resistant *S. aureus* (MRSA) strains (Choi et al., 2015). Jelleines are a family of very small peptides (8-9 amino acid residues long) with +2 positive charge, which are isolated from the royal jelly of honey bees (Fontana et al., 2004) (Figure 2A). Jelleines I-III possess antimicrobial properties against yeast, fungi, gram-positive and gram-negative bacteria (Fontana et al., 2004; Jia et al., 2018).

Magainins are a family of small Gly rich cationic AMPs which are isolated from the skin of African clawed frog *Xenopus laevis* (Figure 2B). They are active against a large number of bacterial and fungal species and cause osmotic lysis of the protozoa. Magainins form amphipathic α-helical conformations in membrane lipid environments. Esculetins are a family of cationic AMPs (46 amino acids long) isolated from frog skin possessing broad-spectrum antimicrobial properties (Simmaco et al., 1994; Ponti et al., 1999). Microbial membranes are the major target of this class of AMPs (Mangoni et al., 2006). Esculentin-2CHa is a 37 residue cationic antimicrobial peptide isolated from the skin of amphibians like *Lithobates chiricahuensis* (Conlon et al., 2011). This peptide is predominantly α-helical in structure with broad-spectrum antimicrobial properties (Vasu et al., 2017a) and low cytotoxicity. Temporins are yet another family of very small α-helical cationic AMPs, with low net positive charge, that are isolated from frog skin (Mangoni, 2006). They are highly potent against a wide range of pathogens like bacteria, fungi, viruses, yeasts and protozoa. Temporins are effective against gram-positive bacterial strains like MRSA and vancomycin resistant *E. faecium* and *Enterococcus faecalis* (*E. faecalis*) (Giacometti et al., 2005). They are non-cytotoxic to mammalian cells and preserve their activity in the presence of serum (Mangoni et al., 2005). Nigrocin 1 and 2 isolated from the skin of *Rana nigromaculata*, manifested broad-spectrum antimicrobial activity against various microorganisms through membranolytic mode of action (Park C. H. et al., 2001). Buforin II, a 21 amino acid residue long cationic AMP was derived from another less active AMP called Buforin I, isolated from Asian toad *Bufo bufo garagrizans* (Park et al., 1996). Buforin II has broad-spectrum antimicrobial activity. Buforin II is non membranolytic and has a very strong affinity for the DNA and the RNA present in the cells (Park et al., 1998). Buforin II has a helix-hinge-helix structure with the hinge at Pro11 residue. N terminal helix contains residues 5–10, while the C terminal helix spans across residues 12–21. Systematic structure activity studies revealed that the α-helical content of the peptide is critical for the antimicrobial potency of Buforin (Yi et al., 1996). Additionally, Pro hinge is extremely important for the cell-penetrating ability of the peptide. Mutation of this Pro converted the peptide into a regular membrane active AMP (Park et al., 2000).

Cathelicidins are a class of human AMPs which are basic in nature. The mature protein was isolated from the neutrophils (Gudmundsson et al., 1996), human skin (Yamasaki et al., 2006) and sweat (Murakami et al., 2002). The peptide contains 37 amino acids and starts with a di-Leucine (Leu) (LL) unit and hence is called LL-37 peptides. LL-37 is active against a whole range of bacteria like *Escherichia Coli (E. coli), S. aureus, P. aeruginosa* and others. Hepcidins are cationic AMPs, 20–25 residues long with low positive charges and are highly disulfide bridged. Human Liver expressed AMP (LEAP-1) was discovered from human blood filtrate (Krause et al., 2000) and urine (Park S. et al., 2001) and was named as Hepcidin 25. It is involved in the iron homeostasis in the body (Pigeon et al., 2001). AMP LEAP-2 is 40 amino acid residues long and does not have any role in iron regulation of the body (Krause et al., 2003).

Some human proteins are antimicrobial in nature. Lysozyme, a positively charged enzyme that is present in animals, forms part of the innate immune system. Lysozyme is a glycosidic hydrolase that catalyses the hydrolysis of 1-4 β-linkages in between the N acetyl muramic acid and N acetyl D glucosamine residues in peptidoglycan that forms active components of the gram-positive bacterial cell wall. This hydrolysis compromises the integrity of the cell wall and causes lysis of the cell. Lysozyme is present abundantly in the secretions like tears, saliva, mucous, human milk, in macrophages and in neutrophils (Manchenko, 1994). Five of the eight ribonucleases have antimicrobial activity. RNase 2 and RNase 3 are both cationic proteins which have anti-parasitic (Hamann et al., 1990) and antiviral activity (Domachowske et al., 1998). RNase 3 also possesses bacterial agglutinating property (Pulido et al., 2013). RNase 7 isolated from human skin (Harder and Schroder, 2002) is active against bacteria and yeast even at 4°C (Huang et al., 2007) and is one of the most abundant innate defense peptide present in the urinary tract (Spencer et al., 2013). RNase 5 is found to have toxic effects against gram-positive bacteria and fungi (Hooper et al., 2003).

### Cyclic Antimicrobial Peptides

**FIGURE 3:**
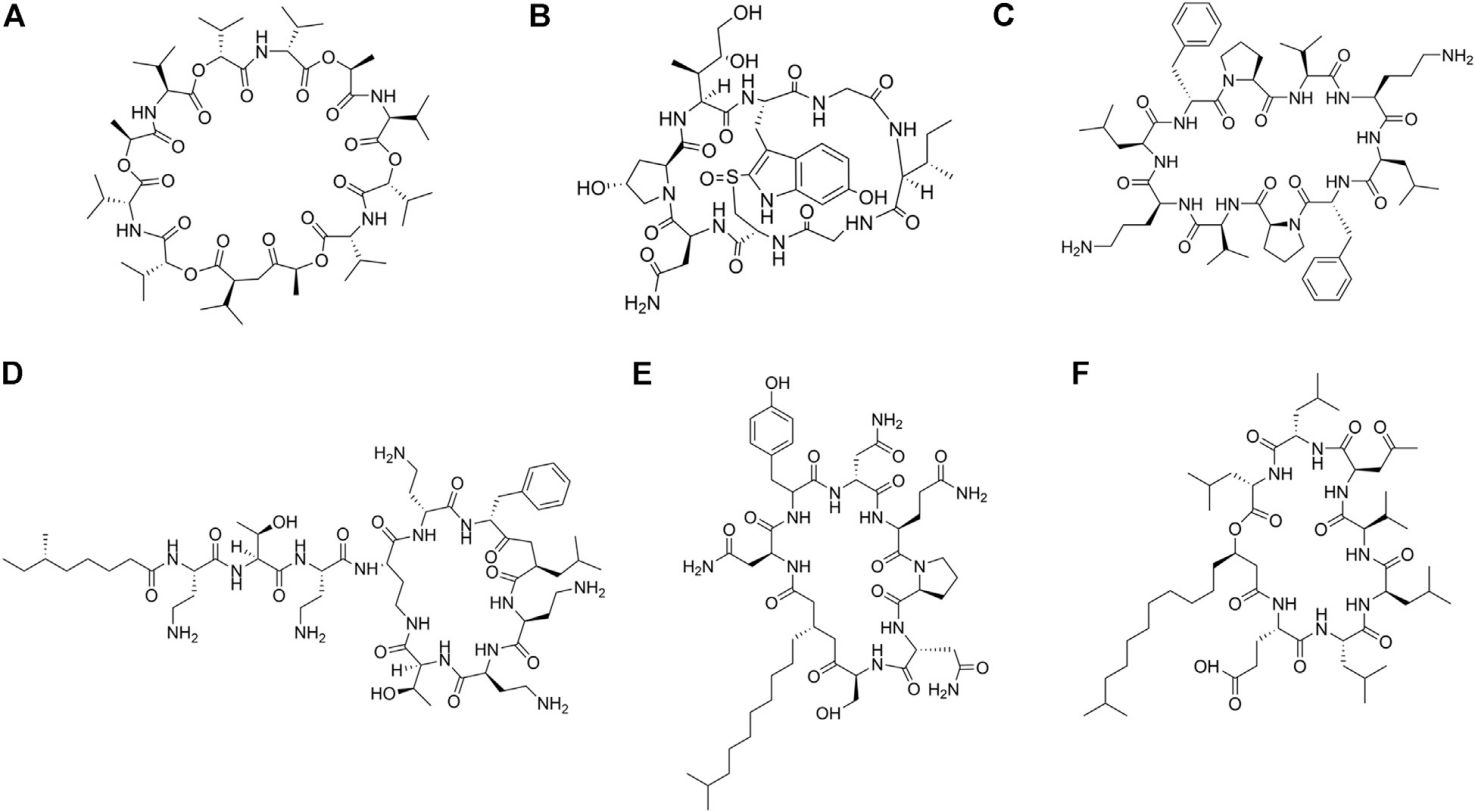
Examples of natural cyclic AMPs. **(A)** Valinomycin. **(B)** Alpha-Amanitin. **(C)** Gramicidin S. **(D)** Polymyxin B. **(E)** Iturin A. **(F)** Surfactin C.

**FIGURE 4:**
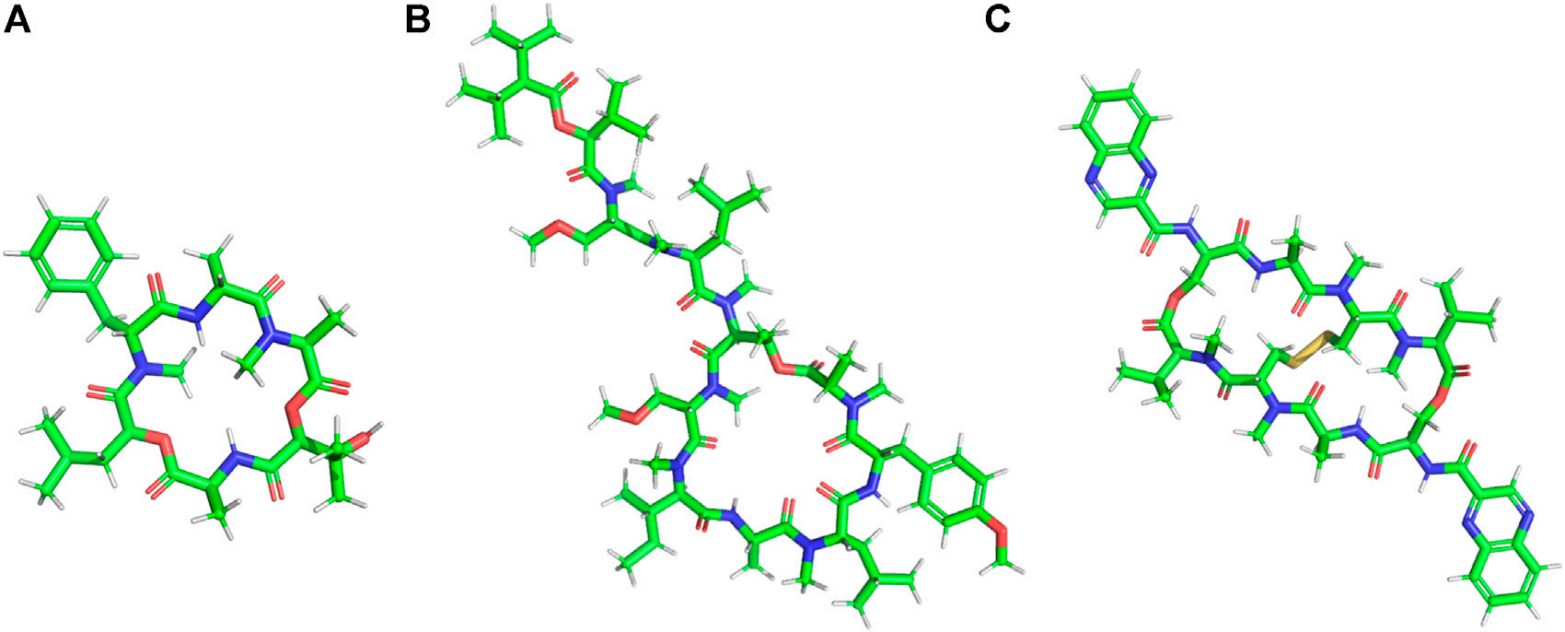
Different modes of cyclization in cyclic AMPs. **(A)** Homodectic cyclic peptides (head-to-tail cyclized): Guangomide A. **(B)** Heterodectic cyclic peptide (head/tail-to-side chain or side chain-to-side chain cyclized): Coibamide A. **(C)** Complex cyclic peptide: Triostin A.

Cyclic AMPs derived from microbes are produced by non-ribosomal pathways by non-ribosomal protein synthetases (NRPs). They contain both proteinogenic and unnatural amino acid residues in them. Functional diversity of these molecules are enhanced by the introduction of various chemical modifications, some of which are epimerization, N-methylation, and heterocyclization (Hur et al., 2012). A large number of cyclic peptides have been isolated from natural resources (Figure 3; Table 1). Cyclic AMPs disrupt the structural integrity of the cells, by disrupting the membrane or targeting the biosynthesis of components of the microbial cell envelope like chitin, glucan, mannoproteins and sphingolipids. Some cyclic AMPs bind to the membrane and cause membranolysis, efflux of intracellular materials and cell death, while others bind to the membrane, traverse them and act upon specific membrane bound structures like ion channels, transporters and receptors. Natural cyclic peptides can be classified depending on the mode of cyclization (Figure 4) or their physicochemical properties. Homodetic cyclic AMPs are formed from the cyclization in between the terminal amino group and the carboxylic acid group. Argyrin B, guangomide, valinomycin (Figure 3A) are some representative examples of homodetic cyclic AMPs. Heterodectic cyclic AMPs are formed from the cyclization in between the side chains of two amino acid residues or in between side chain of one amino acid residue and a terminal amino/carboxylic acid group. Coibamide A, didemnin B, selenamide, luzopeptin are some of the representative examples of this class. Complex cyclic peptides are formed from the mixture of homodetic and heterodetic linkages and may be bicyclic in nature as in the case of amatoxins, amanitin (Figure 3B), phalloidin and triostin A (Lee and Kim, 2015). Cyclic peptides can be charged as in gramicidine S (Figure 3C), polymyxin B (Figure 3D) and bactenecin, non-polar as in argyrin B, aureobasidin and guanomide A, of mixed polarities as in kahalilide F, largamide and pseudodesmin A or containing cyclic cysteine knot (CCK) like kalata B1.

Cyclic Lipopeptides (CLPs) contain a fatty acid tail attached to a cyclic peptide skeleton. These peptides possess broad-spectrum antifungal and antibiotic properties. They mostly act through membrane integration and formation of pores, thereby causing the leakage of the intracellular components and causing eventual cell death. Syringomycins and syringopeptides derived from *Pseudomonas syringae*, possess strong inhibitory activity against the growth of gram-positive bacteria (Grgurina et al., 2005), mycelia (Lavermicocca et al., 1997) and antagonistic property against apple scab causing agent (Burr et al., 1996). Lipodepsipeptide Tolaasin D derived from *Pseudomonas tolaasi* prevents growth of *Rhizoctania solani* and gram-positive bacteria *Rhodococcus fascians* (Bassarello et al., 2004). Gram-negative bacteria *Pseudomonas fluorescence* produces several classes of cyclic AMPs. Pseudophomins A and B produced by *pseudomonas fluorescens* BRG100 significantly inhibit the growth of *Phoma lingam, Alternaria brassicae* and *Sclerotinia sclerotiorum* (Pedras et al., 2003). Massetolide A produced by *P. fluorescens* SS101 possesses a biosurfactant property that affects plant pathogenic oomycetes. Amphisin, lokisin, tensin, and viscosinamide produced by *P. fluorescens* spp. possess *in vitro* antagonistic activities against the oomycete *Pythium ultimum* and the basidiomycete *Rhizoctonia solani* (*R. solani*) (Nielsen et al., 2002; Nielsen and Sorensen, 2003). *Bacillus* species are major producers of CLP’s. Fusaricidins, iturins (Figure 3E), fengycins, polymixins, and agrastatins have been identified in culture extracts of several species of *Bacillus* (Stein, 2005). These CLPs are composed of seven (surfactins (Figure 3F), iturins) or ten (fengycins) α-amino acids linked to one unique β-amino (iturins) or *β*-hydroxy (surfactins, fengycins) fatty acid. The carbon numbers of β-amino fatty acid chain in surfactins, iturins, and fengycins are C_13_-C_16_, C_14_-C_17_, and C_14_-C_18_, respectively. Multiple derivatives of each lipopeptide family are usually co-produced by a strain (Leenders et al., 1999; Akpa et al., 2001). Iturins have antifungal activity against plant pathogens by pore formation in the cell membranes, causing an efflux of potassium and other vital ions eventually causing cell death (Thimon et al., 1992). Fengycin type of cyclic peptides produced by *B. subtilis* (*Bacillus subtilis*) M4 prevents the mycelial growths of *Fusarium oxysporum*, *R. solani* and *B. cinerea* and prevents grey mold disease of apples caused by *B. cinerea* during storage (Ongena et al., 2005). *B. subtilis* CMB32 produces iturin A, fengycin and surfactin A, which are able to suppress the growth of *Colletotrichum gloeosporioides,* the causative agent of anthracnose in a variety of crops (Kim et al., 2010). Surfactins produced by the *Bacillus subtlis* are one of the most effective bio-surfactant known till date and is known to have strong antimicrobial activity against *C. albicans*, *Lactococcus lacti* subsp. *Lactis*, *Mycobacterium smegmatis*, *S. aureus*, and *Streptococcus mutant* (Peypoux et al., 1999; Singh and Cameotra, 2004). Balliomycin produced by *Bacillus species* contains seven α-amino acids and β-amino fatty acids with 14–16 carbon atoms and can control a broad range of plant diseases. Mycosubtilin are heptapeptides containing alternating D and L amino acid residues closed by a β amino acid linkage. *B. subtilis* strains overproducing mycosubtilin are effective in controlling *Pythium* damping off on tomato plants than the parent strain (Leclère et al., 2005). Polymixin B produced by the *Paenibacillus sp*. strain B2 have antifungal activity against root pathogenic fungus *Fusarium solani* and *Fusarium acuminatum* (Selim et al., 2005). Fusaridins are a group of depsipeptides produced by the *Paenibacillus polymyxa* E681 that control the *Phytopthora* blight infection in red pepper caused by *Phytopthora capsica* (Lee et al., 2013). Antibiotic Agrastatin A produced by *B. subtilis* AQ713 has a broad fungicidal spectrum *in vitro* (Heins et al., 2000). Gramicidin S produced by *Brevibacillus Brevis* has both antifungal activity and sporicidal activity (Murray et al., 1986). Daptomycin is a CLP antibiotic that is isolated from soil bacteria *Streptomyces roseosporus*. It is used in treating life threatening infections caused by gram-positive bacteria including MRSA and vancomycin resistant *Enterococci*. Daptomycin has a unique mechanism of action that leads to the destruction of membrane potential (Steenbergen et al., 2005). It is commercially available as a peptide antibiotic drug. Several cyclopeptides are produced by *Streptomyces* species. Valinomycin, first identified from *Streptomyces griseus* has insecticidal, acaricidal (Heisey et al., 1988), antifungal (Park et al., 2008) and antioomecyte activity (Lim et al., 2007). It acts as an ionophore and modulates the transport of potassium ions across biological membranes.

Some natural cyclic AMPs block the biosynthesis of the cellular components. Chitin is a linear homopolymer of β-(1,4)-linked *N*-acetylglucosamine (GlcNAc) residues, and a major component of the fungal cell walls. Polyoxins and Nikkomycins are nucleoside peptide antibiotics that are competitive inhibitors of chitin synthase (Isono and Suzuki, 1979). Arthrichitin is a cyclic depsipeptide that inhibits chitin biosynthesis and works as an antifungal agent (Vijayakumar et al., 1996). Thiopeptide antibiotics are an important class of natural products that are formed from the post‐translational modifications of the ribosomally synthesized peptides. Cyclothiazomycin is a typical example that has a unique bridged macrocyclic structure derived from an 18 amino acid residue peptide (Wang et al., 2010). Cyclothiazomycin B1 produced by *Streptomyces* species acts as an antifungal cyclic thiopeptide that binds to chitin in the fungal cell wall and disrupts the crystallization of the chitin chain. This mechanism inhibits the growth of filamentous fungi and causes swelling of the hyphae and the spores (Mizuhara et al., 2011). Glucans are homopolymers of glucose that form a network of fibrils around the fungal cell that helps to maintain the mechanical strength and the osmotic resistance of the fungal cell wall (Smits et al., 1999). The enzyme β-(1,3)-glucan synthase catalyzes polymerization of glucan. Neopeptins, produced by *Streptomyces* species have potent antifungal properties inhibiting mannoprotein, proteoheteroglycan and β-1-3-glycan synthases found in Sacharomyces cerevisiae (Satomi et al., 1982). Growth of several microbes like *Glomerella cingulata*, *Pleomorphomonas oryzae* (*P. oryzae*), *Cochliobolus miyabeanus,* and *Colletotrichum lagenaium* can be completely inhibited by neopeptins. Aureobasidins are cyclic depsipeptide antifungal antibiotics that are produced from *Aureobasidium pullulans* (Takesako et al., 1991). These are composed of eight lipophilic amino acid residues and one α-hydroxy acid (Ikai et al., 1991a), Ikai et al., 1991b)) and are effective against a large range of fungi and protozoa. Aureobasidin A inhibits inositol phosphorylceramide (IPC) synthase, a key enzyme catalyzing sphingolipid synthesis in fungi.

Cyclotides are groups of homodectic circular proteins that are isolated from plants and animals (Pelegrini et al., 2007; Craik, 2010). Cyclotides have high sequence similarity. They contain three intramolecular disulfide bridges arranged in the form of a CCK(Colgrave and Craik, 2004). The CCK provides cyclotides with exceptional stability towards various proteolytic and degradative processes (Ireland et al., 2010). Kalata B8 isolated from *Oldenlandia affins* (Pelegrini et al., 2007) has a diverse range of biological activities, including uterotonic, anti-HIV, antimicrobial, insecticidal, antihelmintic, and molluscidal properties (Craik, 2010). Kalata B1 interacts with the phosphotidylethanolamine phospholipid present in the membranes leading to membrane bending and vesicle formation. Hevein is a small Cys rich chitin binding protein that is present in the lutoid bodies of the rubber tree (Van Parijs et al., 1991), that inhibits the hyphal growth of fungi. Different hevein like proteins with antimicrobial properties have been isolated from other plants (Koo et al., 1998; Kiba et al., 2003; Huang et al., 2004; Porto et al., 2012). These peptides are small, 20–40 amino acids long, rich in Cys and Gly residues at conserved positions. Hevein like peptides have eight (Li and Claeson, 2003), six (Huang et al., 2000) or ten (Huang et al., 2002) Cys residues in them, giving rise to conserved disulfide bridges.

Ranacyclins E and T are 17 residue cyclic peptides from amphibians that exhibit antimicrobial and antifungal activity (Mangoni et al., 2003a). Theta (θ) defensins found in non-human primates are18 residue cyclic peptides formed by head-to-tail cyclization of the peptide (Tang et al., 1999; Selsted, 2004). Like the α and the β-defensins, this class of peptides contain three disulfide bridges in between the Cys I-IV, Cys II-V and Cys III-IV. These genes are not expressed in humans due to the presence of a premature stop codon. Like monkey θ defensins, synthetic versions of the human analogs have anti-HIV activity (Cole et al., 2004; Yang et al., 2005).

### Antimicrobial Peptides rich in specific amino acid residues (Tryptophan/Proline/Histidine/Glycine)

**FIGURE 5:**
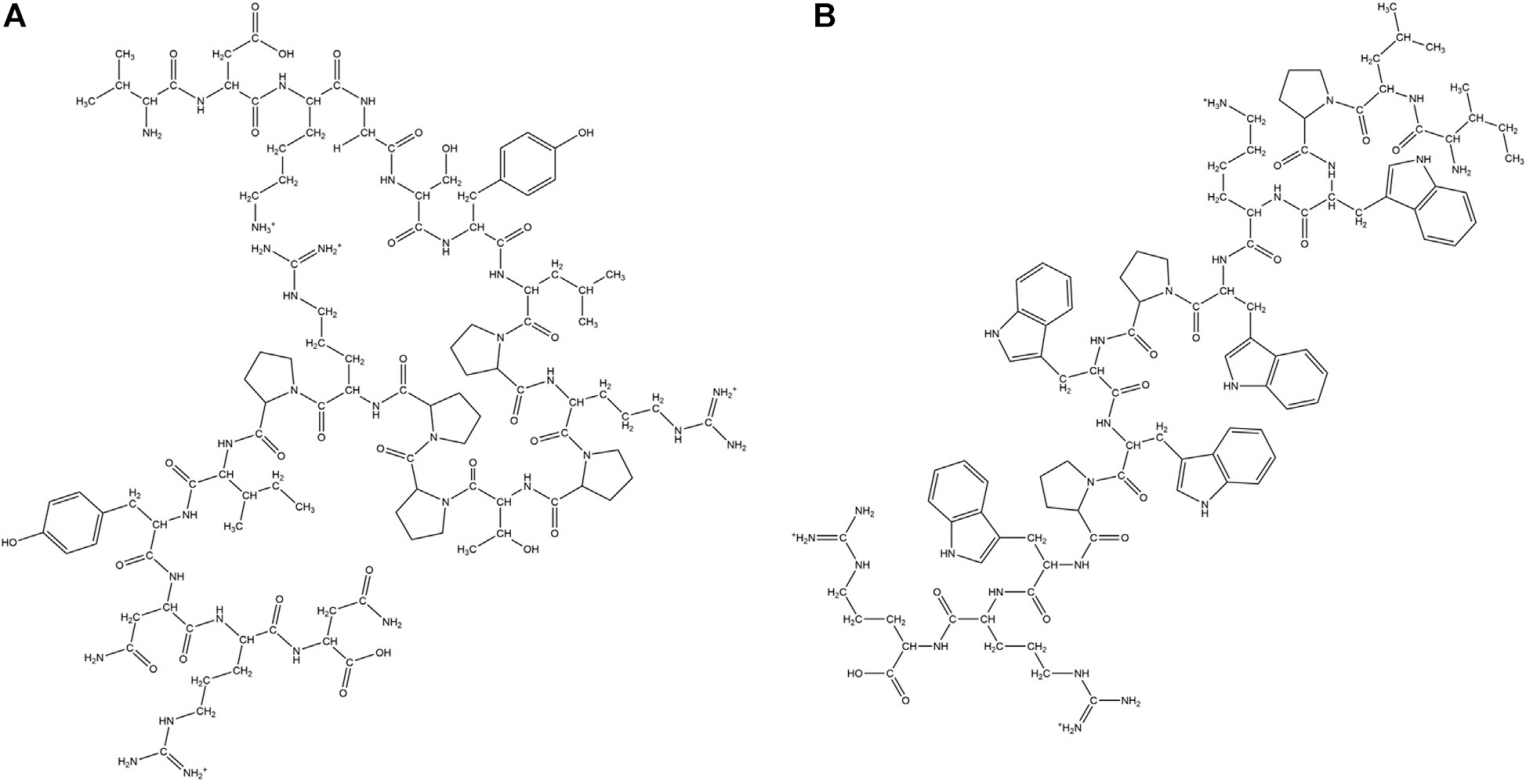
Examples of natural AMPs rich in Pro and Trp amino acid residues. **(A)** Pro rich AMP: Pyrrhocoricin. **(B)** Trp rich AMP: Indolicine.

Many AMPs have very high content of a certain amino acid residues like Trp (Chan et al., 2006; Dong et al., 2012), Pro (Kragol et al., 2002), Gly (Baumann et al., 2010; Ili´c et al., 2013) or His (Oppenheim et al., 1988) (Figure 5; Table 3). These AMPs are usually extended having no regular structure and may be cationic or anionic in nature. Many of these peptides are known to have membrane permeability and intracellular targets.

Trp residues are found in high proportions in a sub-class of short peptides (TrAMPs) (Shagaghi et al., 2016). The Trp residue usually appears near the C terminal end of the peptides (Schibli et al., 2002). TrAMPs are also usually Arg rich. Most of the TrAMPs permeate bacterial membranes and acts on intracellular targets without disrupting membrane integrity. Only a very few like Indolicidin have membrane-disruptive activities. These peptides are active against different species of bacteria and fungi.

Lactoferricins are mammalian TrAMPs, obtained from the peptide mediated digestion of lactoferrins which exhibit antimicrobial (against bacteria, fungi and viruses) (Wakabayashi et al., 2003; Gifford et al., 2005) and anticancer activity (Eliassen et al., 2002). Lactoferricins are cationic, amphipathic in nature. While the bovine lactoferricin is 25 amino acid residue long and mostly forms β-sheets (Hunter et al., 2005), the human lactoferricin is 49 residues long and forms coiled coils (Gifford et al., 2005). Indolicidin is a 13 residue (ILPWKWPWWPWRR-NH2) cationic TrAMP isolated from bovine neutrophils (Figure 5A). Indolicidin has broad-spectrum activity against bacteria, fungi, protozoa and virus (Subbalakshmi and Sitaram, 1998). It is also active against MRSA, HIV virus (Robinson et al., 1998) and herpes simplex virus. The peptide directly inactivates the virus particles without being cytotoxic to the Vero cell line (Albiol Matanic and Castilla, 2004). Tritrpticin is another 13 amino acid residue long Tr-AMP that is derived from a precursor protein in the porcine bone marrow with high homology to the indolicidins. They have bactericidal and fungicidal activities against several clinical microorganisms (Lawyer et al., 1996).

Pro rich AMPs (Pr-AMPs) usually have conserved repeats of Pro and Arg. They do not adopt amphipathic structure but β chain type polyproline or cyclic helix structure instead, as in Nisin and PR-39. Pr-AMPs are cationic peptides expressed in arthropods as well as animals. They have low sequence homology but high structural homology of about 30% Pro content and several Pro-Arg-Pro motifs. They usually kill bacteria by non-lytic mechanisms. Pro rich peptides like Drosocin (Bulet et al., 1993), Pyrrhocoricin (Figure 5B) (Gagnon et al., 2016), Metalnikowin 1 (Gagnon et al., 2016) are known to inhibit protein biosynthesis by binding to the 70S ribosomal subunit, inhibiting heat shock proteins like DnaK and GroEL and binding to the lipopolysaccharides (LPS). In general Pro rich peptides enter into the cell through translocation of the inner membrane *via* a transporter mediated uptake mechanism by membrane protein SbmA (Runti et al., 2013). Several other Pr-AMPs like gonocin, apedaicin, drosocin are known to cross the blood brain barrier (Lalatsa et al., 2014; Stalmans et al., 2014). Apidaecins are the first class of Pr-AMPs that were isolated from honey bees. The mature peptide is linear, 19 residues long, heat stable and non-helical in nature. They are known to be active against a large number of plant associated bacteria and human pathogens through a bactericidal pathway rather than a lytic process (Casteels et al., 1989). Abaecin is a 32 amino acids long Pr-AMPs, isolated from honeybee *Apis mellifera* with strong antimicrobial and antifungal activity (Casteels et al., 1990). Lebocins are 32 amino acids long glycosylated Pr-AMPs isolated from silkworm *Bombyx mori* (Hara and Yamakawa, 1995; Liu et al., 2000). Lebocins have 41% sequence similarity with the baecin, a 34 residue antimicrobial peptide isolated from honeybee (Casteels et al., 1990). Drosocin is a post-translationally modified PrAMP isolated from *Drosophila melanogaster* (Bulet et al., 1993; McManus et al., 1999). Drosocin contains Pro-His repeats, along with a Threonine (Thr) residue and 5 N terminal residues critical for its activity (Bulet et al., 1993). Drosocin is active against *E. coli* and fungi (Imler and Bulet, 2005). Drosocin binds to bacterial DnaK, inhibiting cellular pathways (Zahn et al., 2013) and microbial ribosomes preventing protein translation (Florin et al., 2017). Pyrrhocoricin is a cyclic PrAMP isolated from *Pyrrhocoris apterus* (Cociancich et al., 1994; Rosengren et al., 2004) that binds and promotes ATPase activity in the heat shock protein DnaK (Kragol et al., 2001; Chesnokova et al., 2004). Closely related to drosocin and abaecin in sequence and function, this acts as a delivery vehicle in transducing peptides across cell membranes of the important human parasite pathogen *Cryptosporidium parvum* (Boxell et al., 2008). Metchnikowin, an immune inducible linear Pr-AMP peptide from *D. megalogaster* is selectively potent against gram-positive bacteria and fungi with no activity against gram-negative bacteria (Levashina et al., 1995). Metchnikowin interacts with fungal enzyme β (1,3)-glucanosyl transferase Gel1, that is involved in the fungal cell wall synthesis (Levashina et al., 1998). Diptericins (8 kDa) constitute a family of Pro and Gly rich AMP from Dipteran hemolymph proteins, which are about 82 amino acids long. Dipterin A is active against a few gram-negative bacteria like *E. coli* K12, *Erwinia hericola* T and *Erwinia carotovora* 113. This AMP acts on the cytoplasmic membrane of the growing bacteria (Keppi et al., 1989). Bacternecin is a mammalian PrAMP that is found in mammalian species like cow, goat and sheep. It is referred to as Bac 5 or 7 depending on the molecular weight of the mature peptides (Gennaro et al., 1989). Bac5 (43 amino acids) and Bac7 (60 amino acids) enter bacterial cells through the SmbA transporter and work on the intracellular targets (Gennaro et al., 2002; Runti et al., 2017). In pigs, Pr-AMP corresponding to the Bac 7 is called PR-39 after the number of constituent amino acids (Agerberth et al., 1991).

Human histatins are His rich AMPs. Histantins 1 and 3 are isolated from human saliva (Oppenheim et al., 1988). Histatin 5 is present in the secretions of human submandibular and parotid glands (Oppenheim et al., 1988). All the three histatins can kill pathogenic yeast *C. albicans*. Calcitermin C is a 15 residue AMP found in human airways and is effective against the gram-negative bacteria. It contains three His residues at the N terminus and a potential to adopt a helical conformation in the membranes (Cole et al., 2001).

Several Gly rich peptides have been isolated from the bacteria, plants, insects, spiders, nematodes, fish and amphibians KDAMP is a keratin derived Gly rich AMP that is present in the bactericidal lysate fractions of the human corneal epithelial cells (Tam et al., 2012). Attacins are Gly rich proteins (10–22%) first isolated from *Hyalophora ceropia* (Hultmark et al., 1983) and effective against gram-negative bacteria (Carlsson et al., 1991). Attacins are subdivided into subclasses A-F. Among these, Attacin A-D constitute the basic group while sub-classes E-F constitute acidic group of peptides. Attacins act via preventing the synthesis of the major outer membrane proteins, that disturb the integrity of the cell wall leading to formation of chains of bacteria (Carlsson et al., 1998). Attacins are mostly random coil in structure owing to the presence of high quantities of Gly residues. Persulcatusin is a Gly rich (20%) cationic AMP that is isolated from the mid-gut region of tick *Ixodes persulcatus* (Saito et al., 2009). This peptide adopts both α-helical and β-sheet structures that is stabilized by three disulfide bonds (Miyoshi et al., 2016, 2017). Persulcatusin can inhibit the growth of Methycilin sensitive *S. aureus* (MSSA) and Methycilin resistant *S. aureus* (MSRA) (Mylonakis et al., 2016) and multi drug resistant (MDR) *S. aureus* strains (Miyoshi et al., 2017). Ponericins are another family of Gly rich peptides isolated from the venom of the predatory ants. Ponericins are sub-divided into three classes depending on the primary sequence of the peptides. Ponericin G have sequence similarity with cecropin kind of peptides, ponericin W resembles melittin and ponericin L is similar with dermaceptins. They have α helical structures in cell membranes. Ponericins show hemolytic and insecticidal activities against cricket larvae.

## Drawbacks of Natural Antimicrobial Peptides

In spite of a very large repertoire of natural AMPs identified, isolated and characterized from various classes of living organisms, there is only a limited success in the commercialization of these peptides. This is due to several drawbacks of the natural AMPs.

1) Long AMP sequences are economically and synthetically challenging for pharmaceutical scale synthesis.
2) AMP sequences often contain complex disulfide bridges and CCKs which are challenging to be synthetically mimicked.
3) Folding of synthetic AMPs into secondary structures (α-helical or β-sheet), critical for their activity, may be challenging, leading to their inactivity.
4) Many AMPs that are produced in the other organisms may show toxicity and high immunogenicity when administered in humans, making them therapeutically inappropriate.
5) Natural AMPs have short serum half-life due to their susceptibility to the protease degradation and physiological salt concentrations.
6) Site directed delivery of AMPs to the site of infection is challenging.

To overcome the shortcomings of the natural AMPs, there has been a surge of interest in generating synthetic AMPs. Several approaches have been adopted for the design of synthetic peptides as discussed below.

1) Template based synthesis: Involves *de novo* sequencing of the synthetic peptides based on natural AMP sequences to improve their activity, cytotoxicity, proteolytic stability and salt tolerance This method includes truncation of sequences, mutation of specific amino acid residues, enrichment of primary sequence with certain types of residues, altering of the charge, hydrophobicity, secondary structure of the designed peptides, fusion of different peptide sequences, incorporation of unnatural amino acid residues, D amino acid residues, peptidomimetic fragments, cyclization, lipidation etc. with an intension to improvise on the shortcomings of the template natural AMP sequence. This is the most convenient and commonly practiced way of synthetic AMP design.
2) Rigorous biophysical modelling: In this process, the prospective synthetic peptides are first modelled and are studied in hydrophobic membrane environments to have an atomistic idea of the designed peptides and their activities.
3) Virtual screening: Virtual screening of the library of existing peptides is done to identify potential AMP sequences. This screening may be based on certain conserved primary sequence elements or secondary structural elements.

Truncation of the sequences introduces economic viability and facility in synthesis without compromising the antimicrobial potency. Improvement in the antimicrobial potency is attempted by mutation of/enrichment with certain amino acid residues, increase of the net charge/hydrophobicity of the peptide, optimization of the hydrophilic/lipophilic balance etc. Protease resistance of the AMPs is achieved by introducing chemical modifications like cyclization, terminal protection (Jenssen, et al., 2006) and incorporation of D-amino acid residues (Vunnam et al., 1998; Chen et al., 2006; Jung et al., 2007) unnatural amino acid residues (Lu et al., 2020) or peptidimimetic fragments. For efficient *in vitro* delivery, nanostructured AMP conjugates are designed. Hydrogels (Borro et al., 2020) and nano-assemblies based on metal/metal oxide nanoparticles (Pal et al., 2019) are being explored recently as prospective methods of AMP delivery.

## Synthetic Antimicrobial Peptides

In this section, we are going to describe some representative examples of synthetic AMPs which are cationic, cyclic and rich in amino acid residues like Trp, Pro, His and Gly (Table 2).

**TABLE 2:** Synthetic antimicrobial peptides (aa = amino acid, res = residues).

Name of the peptides		Sequence of the peptides	Physiochemical properties	Mechanism of action	Target microbes	References
Synthetic cationic antimicrobial peptides						
(KIGAKI)_3-_NH_2_		KIGAKIKIGAKIKIGAKI-NH2	18 aa res; +7 net charge; Lys rich (33%)	Membranolytic; Intracellular mode of actions expected as well	*S. aureus*, *E. coli*, *P. aeruginosa*	Blazyk et al. (2001)
V681n		KWKSFLKTFKSAVKTVLHTALKAISS	26 aa res; +6 net charge; Lys rich (23%)	Possibly membranolytic	*E. coli*, *P. aeruginosa*	Zhang et al. (1999)
V26p		KWKSFLKKLTSAAKKVLTTALKPISS	26 aa res; +7 net charge; Lys rich (27%)	Possibly stimulation of autolytic enzymes, interference with bacterial DNA and/or inhibition of protein synthesis	*E. coli*, *P. aeruginosa*	Zhang et al. (1999)
JH8194		KRLFRRWQWRMKKY	14 aa res; +7 net charge; Arg rich (29%), Lys rich (21%)	-	*C. albicans*	Nikawa et al. (2004)
D8R8 (Dendrimer R8)		(RLYRKVYG)2-[(K2K)2K] (Dendrimeric R8)	64 aa res; +24 net charge; Arg rich (25%)	Membranolytic	*E. coli*, *P. aeruginosa*, *P. vulgaris*, *K. oxytoca*, *S. aureus*, *M. luteus*, *E. faecalis*, *C. albicans*, *C. kefyr*, *C. tropicalis*	Tam et al. (2002)
Peptide 20		[(2-Cl-Z)-K-(HCl)]2-KF-NH(CH2)11CH3	Lys rich	Inhibition of the activity of candidal β-(1,3)-glucan synthase, a membrane-located enzyme	*S. aureus*, *E. coli*, *P. aeruginosa*, *B. bronchiseptica*, *K. pneumonia*, *A. baumanii*, *C. albicans*, *C. krusei, C. parapsilosis*	Janiszewska et al. (2012)
Esculentin-1b (1–18)		GIFSKLAGKKLKNLLISGL-NH2	19 aa res; +5 net charge; Lys rich (21%)	Membranolytic	*E. coli*, *P. aeruginosa*, *Y. pseudotuberculosis*, *E. faecalis*, *E. agglomerans*, *B. megaterium*, *S. aureus*, *S. pyogenes*, *S. epidermidis*, *S. haemolyticus*, *S. hominis*, *S. capitis etc.*	Mangoni et al. (2003b)
Esculentin-1a (1–21)		GIFSKLAGKKIKNLLISGLKG-NH2	21 aa res; +6 net charge; Lys rich (23%)	Membrane permeabilization	*S. cerevisiae*	Gamberi et al. (2007)
(Aib1,10,18)-Esc (1–21)		Aib-IFSKLAGK-Aib-IKNLLIS-Aib-LKG-NH2	21 aa res; +5 net charge; Lys rich (19%)	-	*A. baumannii, E. coli*, *P. aeruginosa*, *Y. pseudotuberculosis*, *B. megaterium*, *S. aureus*, *S. capitis*, *S. epidermidis*, *S. epidermidis*, *S. hominis*, *C. albicans etc*	Biondi et al. (2017)
C12-fXXL		C11-CO-DPhe-Dab-Dab-Leu-NH2 (Dab - Diaminobutyric acid)	4 aa res; +2 net charge; Dab rich (50%)	Membranolytic	*E. coli*, *K. pneumonia*, *P. aeruginosa*, *Y. enterocolitica*, *E. faecalis*, S*. aureus*, *S. epidermidis*	Juhaniewicz-Dębińska et al. (2020)
003c		Gpp-Bip-Gpp	3 aa res; +3 net charge	-	*S. aureus*, *Methicillin-resistant S. aureus*	Svenson et al. (2009)
Peptide 3		R-Bip-R-NHBn	3 aa res; +3 net charge; Arg rich (66%)	-	*S. aureus*, *Methicillin-resistant S. aureus*	Karstad et al. (2010)
TAL L512		NH2-FLPLLGRVLSGLL-CONH2	13 aa res; +2 net charge; Leu rich (46%)	-	*S. aureus*	Mantyla et al. (2005)
S8		C_7_H_15_-CO-dgF-K-dgF-K-NH_2_	4 aa res; + 2 net charge; dgPhe rich (50%), Leu rich (50%)	Disruption of outer and inner membranes	*E. coli*, *K. pneumonia*, *P. aeruginosa*, *S. aureus*, *S. typhimurium*, *C. albicans*, *C. glabrata*, *C. neoformans*, *S. cerevisiae*	Shankar et al. (2013)
(KLAKKLA)3		KLAKKLAKLAKKLAKLAKKLA	21 aa res; +9 net charge; Lys rich (43%); Leu rich (29%)	-	*E. coli*, *P. aeruginosa*, *S. aureus*	Javadpour et al. (1996)
HPA3NT3-analog		FKKLKKLFKKILKLK-NH2	15 aa res; +9 net charge; Lys rich (53%)	Membranolytic	*E. coli*, *S. typhimurium*, *P. aeruginosa*, *S. aureus*, *B. subtilis*, *L. monocytogenes*, *C. albicans*, *T. beigelli*	Gopal et al. (2009)
Peptide 3		KW-K(Me3)-LFKKIGAVLKVL-NH2	15 aa res; +5 net charge; Lys rich (33%)	Membrane permeation	*S. aureus*, *A. baumannii*, *L. donovani promastigotes*, *L. pifanoi amastigotes*	Fernandez-Reyes et al. (2010)
H-DS-5W		H-KXAKWXKKXAKAXAK-NH2 (cross-linked by two oct-4-enyl staples, each represented by two X-X, three amino acids apart)	11 aa res; +7 net charge; Lys rich (55%)	-	*B. subtilis*, *S. aureus*, *S. epidermis*, *E. coli*, *S. dysentariae*, *S. typhimurium, K. pneumonia*, *P. aeruginosa*	Dinh et al. (2015)
P4		LKWLKKL-NH2	7 aa res; +4 net charge; Lys rich (43%), Leu rich (43%)	Membranolytic	*E. coli*, *P. aeruginosa*, *K. pneumoniae*, *S. aureus*, *C. albicans*, *C. grubii*	Pandit et al. (2018)
AMP 22		LRKVWRWLRRL-NH2	11 aa res; +6 net charge; Arg rich (36%)	Membrane perturbation as well as intracellular secondary mode of action	*E. coli*, *P. aeruginosa*, *K. pneumoniae*, *S. aureus*, *C. albicans*, *C. grubii*	Pandit et al. (2020)
VV-14		VKWVKKVVKWVKKV-NH2	14 aa res; +7 net charge; Val rich (43%), Lys rich (43%)	Membrane deformation, depolarization, and lysis	*E. coli*, *P. aeruginosa*, *S. typhi*, *S. aureus*, *K. pneumoniae*, *C. albicans*, *C. neoformans*, *C. grubii*	Pandit et al. (2021)
Triblock amphiphilic peptide (K_3_F_6_K_3_)		KKKFFFFFFKKK	12 aa res; +6 net charge; Lys rich (50%)	Membranolytic	*E. coli*, *S. aureus*	Huang et al. (2020)
PR-9		PFWRIRIRR-NH2	9 aa res; +5 net charge; Arg rich (45%)	Intracellular	*E. coli*, *P. aeruginosa*, *S. aureus*, *S. pyogenes*, *E. faecalis*, *E. aerogenes*, *E. cloacae*, *B. megaterium*	Epand et al. (2010)
capA_6_R		CH3-CONH-A6R-NH2	7 aa res; +1 net charge; Ala rich (86%)	Membranolytic	*S. aureus*, *L. monocytogenes*, *E. coli*	Castelletto et al. (2018)
C_12-_SC4		C12-KLFKRHLKWKII-NH2	12 aa res; +5 net charge; Lys rich (33%)	Destabilization of bacterial membranes	*E. coli, S. epidermidis*	Chu-Kung et al. (2004)
NB-119–2		C_16_-(α-AA)_5_-NH_2_	5 aa res; +5 net charge	Membranolytic	*B. subtilis*, *S. epidermidis*, *E. faecalis*, *S. aureus*, *E. coli*, *K. pneumoniae*, *P. aeruginosa*, *C. albicans*	Hu et al. (2012)
N-C_8_		(C8-K)-RWRWRW-NH2	7 aa res; +4 net charge; Arg rich (43%), Trp rich (43%)	-	*E. coli*, *A. baumannii*, *P. aeruginosa*, *S. aureus*, *S. aureus*, *B. subtilis*	Albada et al. (2012)
Ano- D4,7–7C_10_		GLLkRIk(C10)TLL-NH2 (Small letters indicate D-amino acids)	10 aa res; +4 net charge; Leu rich (50%)	Membranolytic as well as intracellular	*E. coli*, *P. aeruginosa*, *A. baumannii*, *K. pneumoniae*, *S. aureus*, *B. subtilis*, *S. epidermidis*	Zhong et al. (2020)
Peptide 79		KL-Tic-Oic-KG-Tic-Oic-FG-Tic-Oic-KG-Tic-Oic-FG-Tic-Oic-KG-Tic-Oic-KKKK-NH2	28 aa res; +9 net charge; Lys rich (29%), Tic rich (18%), Oic rich (22%)	-	*S. aureus*, *A. baumannii*, *K. pneumoniae*, *E. aerogenes*, *E. faecium*	Hicks et al. (2013)
Cbf-14-2		RLLR-Orn-FFR-Orn-LKK-SV-NH2 (Orn)	14 aa res; +7 net charge; Arg rich (22%)	Membranolytic	*S. aureus*, *E. coli*, *P. aeruginosa*, *C. albicans*	Kang et al. (2017)
(RW)_4D_		Dendrimeric peptide with four RW units	Arg and Trp rich	Membranolytic	*E. coli*	Hou et al. (2009)
Antimicrobial β-peptide		H-(β^3^- HVal-β^3^-HLys-β^3^-HLeu)_4_-OH	β aa res containing peptides	Membranolytic	*E. coli*	Liu et al., 2001
Synthetic cyclic peptides						
(Gly_6_)ccTP		(K**G**C**G**R**G**C**G**R**GG**C**G**RRCR**G**) (Tricyclized: cyclized end to tail, through cys at position 3 and 16, and through cys at 7 and 12)	18 aa res; +6 net charge; Gly rich (39%)	Membranolytic	*E. coli*, *P. aeruginosa*, *Pr. Vulgaris*, *K. oxytoca*, *S. aureus*, *M. luteus*, *E. faecalis*, *C. albicans*, *C. kefyr*, *C. tropicals*	Tam et al. (2000)
(R4W4)		-	8 aa res; +4 net charge; Trp rich (50%), Arg rich (50%)	-	*Methicillin-resistant S. aureus*, *P. aeruginosa*	Oh et al. (2014)
C12-(R5)		Dodecanoyl-(KRRRRR)	6 aa res; +5 net charge; Arg rich (83%)	-	*Methicillin-resistant S. aureus*, *P. aeruginosa*	Oh et al. (2014)
RH 11		NH2-(CwWkKkKkWwC)-CONH2	11 aa res; +6 net charge; Lys rich (45%)	Membranolytic	*P. aeruginosa*	He et al. (2017)
Peptide 4		(kQrWlWlW) (Small letters represents D-amino acids)	8 aa res; +2 net charge; Trp rich (38%)	-	*Methicillin-resistant S. aureus*, *E. coli*, *B. subtilis*, *B. cereus*, *S. aureus*, *L. monocytogenes*, *E. faecalis*, *S. pneumonieae*, *S. typhimurium*	Fernandez-Lopez et al. (2001)
Peptide 7		(WlWkUkSk) U = Ser(α-Mannose) (Small letters represent D-amino acids)	8 aa res; +3 net charge; glycosylated	-	*Methicillin-resistant S. aureus*, *Vancomycin-resistant E. faecalis*, *B. cereus*	Motiei et al. (2009)
Synthetic antimicrobial peptides rich in W, P, H, G						
PEP 6	frlkfh (all D amino acids)		6 aa res; +2 net charge	Membranolytic	*F. oxysporum f. sp.*, *L. Rhizoctoni solani*, *C. fagacearum*, *P. ultimum*	Reed et al. (1997)
PAF 26	Ac-rkkwfw-NH2 (Small letters represent D-amino acids)		6 aa res; +3 net charge; Lys rich (33%), Trp rich (33%)	-	*P. digitatum*, *P. italicum*, *B. cioneria*	Lopez-García et al. (2002)
Active center of Lactoferricin B	RRWQWR-NH2		6 aa res; +4 charge; Arg rich (50%), Trp rich (33%)	Membranolytic	*Xanthomonas campestrins pv. Vesicatoria*, *Clavibacter subsp. Michiganensis*, *R. solanacearum*, *E. coli*, *B. subtilis*	Tomita et al. (1994)
KCM 21	KWWWRW-NH2		6 aa res; +3 net charge; Trp rich (67%)	-	*E. coli*, *S. aureus*	Choi and Moon (2009)
(RW)_3_	RWRWRW-NH2		6 aa res; +4 net charge; Trp rich (50%), Arg rich (50%)	Membranolytic	*E. coli*, *S. aureus*, *R. solani*, *R. cerealis*	Liu et al. (2007)
GSP-5D	MPLSWFFPRTWGKR-NH_2_		14 aa res; +4 net charge	-	*E. coli*, *S. aureus*, *R. solani*, *R. cerealis*	Phillips et al. (2011)
GA-K4AL	FAKWAFKWLKK-NH2		11 aa res; +5 net charge; Lys rich (36%)	-	*B. subtilis*, *M. luteus*, *S. aureus*, *S. epidermis*, *E. coli*, *S. dysentariae*, *S. typhimurium*, *K. pneumoniae*, *P. aeruginosa*	Won et al. (2011)
PW2	HPLKQYWWRPSI		12 aa res; +2 net charge	-	*E. tenella*, *E. acervuline*, *C. albicans*, *A. nidulans*	Da silva et al. (2002)
CP-11	ILKKWPWWPWRRK-NH2		13 aa res; +6 net charge; Trp rich (30%), Lys rich (23%)	Both membranolytic and intracellular	*E. coli*, *P. aeruginosa*, *S. typhimurium*, *S. aureus*, *S. epidermidis*, *E. faecalis E. coli*, *P. aeruginosa*, *S. typhimurium*, *S. aureus*, *S. epidermidis*, *E. faecalis*	Rozek et al. (2003)
CycloCP-11	ICLKKWPWWPWRRCK-NH2 (Cyclized through S-S cys bonds at the temini)		15 aa res; +6 net charge; Trp rich (27%), Lys rich (20%)	Both membranolytic and intracellular	*E. coli*, *P. aeruginosa*, *S. typhimurium*, *S. aureus*, *S. epidermidis*, *E. faecalis E. coli*, *P. aeruginosa*, *S. typhimurium*, *S. aureus*, *S. epidermidis*, *E. faecalis*	Rozek et al. (2003)
CP-11C	ILKKWPWWPWRRK-OMe		13 aa res; +5 net charge; Trp rich (30%), Lys rich (23%)	Membranolytic	*E. coli*, *P. aeruginosa*, *S. typhimurium*, *S. aureus, S. epidermidis*, *C. albicans*	Falla and Hancock (1997)
RN7-IN10	FLGGLIKWKWPWWPWRR-NH2		17 aa res; +5 net charge; Trp rich (29%)	Membranolytic as well as inhibition of intracellular functions due to DNA binding	*S. pneumoniae*	Jindal et al. (2017)
Puro A	FPVTWRWWKWWKG-NH2		13 aa res; +4 net charge; Trp rich (38%)	Membranolytic	*E. coli*, *S. aureus*	Jing et al. (2003)
PR 26	RRRPRPPYLPRPRPPPFFPPRLPPRI		26 aa res; +8 net charge; Pro rich (46%), Arg rich (30%)	Membranolytic	*E. coli*, *Salmonella* spp.	Shi et al. (1996)
Bac5 (1–25)	RFRPPIRRPPIRPPFYPPFRPPIRP		25 aa res; +7 net charge; Pro rich (44%), Arg rich (28%)	Intracellular and nonlytic mode of action	*E. coli*	Mardirossian et al. (2018)
GKH17-WWWWW	GKHKNKGKKNGKHNGWKWWWWW		22 aa res; +7 net charge; Lys rich (32%), Trp rich (27%), Gly rich (18%)	Membranolytic	*P. aeruginosa*	Pasupuleti et al. (2009)
PS1-5	(RWYR)3-HN2		12 aa res; +7 net charge, Arg rich (50%)	Membranolytic	*E. coli*, *P. aeruginosa*, *S. typhimurium, S. aureus*, *S. epidermidis*, *L. monocytogens*	Lee et al. (2018)
^4Har^HHC-10	Har-Har-Trp-Trp-Har-Trp-Ile-Har-Trp-NH2 (Har: L-homoarginine)		9 aa res; +5 net charge; Trp rich (44%), Har rich (44%)	Membranolytic	*E. coli*, *S. aureus*	Bagheri et al. (2020)
12b	Dendrimeric (WRW)3		Dendrimeric, trivalent; 3 aa res; +3 net charge; Trp rich (67%), Arg rich (33%)	Membranolytic	*E. coli, A. baumannii, B. subtilis, S. aureus*	Hoffknecht et al. (2015)
-	RWRWRWA-(Bpa)		7 aa res; +4 net charge; Trp rich (42%), Arg rich (42%)	-	*P. aeruginosa*	Mao et al. (2018)
KU 2	GIWKKWIKKWLNVLKNLF-NH2		18 aa res; +5 net charge; Lys rich (28%)	-	*C. albicans*	Lum et al. (2015)
P6 (Modified PMAP-23)	RIIDLLWRVWRPWWPKFVTVWVR-NH2		23 aa res; +5 net charge; Trp rich (22%)	Membranolytic	*C. albicans*	Lee et al. (2002)
RV-23	RIGVLLARLPKLFSLFKLMGKKV-NH2		23 aa res; +7 net charge; Lys rich (18%)	Membranolytic	*E. coli*, *S. aureus*	Zhang et al. (2016)
Di-K19Hc	(KWLNALLHHGLNCAKGVLA)_2_ (Dimerized through Cys)		38 aa res; +6 net charge	Membranolytic	*P. grisea*, *F. oxysporum*, *A. terreus*, *A.niger*, *G. candidum*, *Cr. neoformans*, *T. beigelli*, *C. albicans*	Jang et al. (2006)
(D)-K5L7-UA	KKllKLLlKlLK-NHCO-(CH2)9-CH3 (Small letters signify D-amino acids)		12 aa res; +5 net charge; Leu rich (58%), Lys rich (42%)	Membranolytic	*C. albicans*, *C. neoformans*, *A. fumigatus*, *E. coli*, *P. aeruginosa, B. subtilis, S. aureus*	Avrahami and Shai (2003)
P0-1	GIKKWLHSAKKFGKKFVKKIMNS-NH2		23 aa res; +9 net charge; Lys rich (35%)	Membrane permeabili-zation and DNA binding	*E. coli, S. epidermidis*	Azuma et al. (2020)

### Synthetic Cationic Antimicrobial Peptides

As cationic AMPs are the most abundant type of AMPs in nature, a lot of synthetic designs are based on cationic sequences. Blazyk et al*.* designed linear cationic peptides to demonstrate that amphipathicity in secondary structure was more important than the kind of secondary structure. (KIGAKI)_3_ which formed amphipathic β-sheet was more potent than (KIAGKIA)_3_ which formed a non-amphipathic α-helix upon membrane binding (Blazyk et al., 2001). In a subsequent study, two families (α-helices and β-sheets) of synthetic linear cationic peptides with same charge and C terminal amidation were studied. Only peptides able to form highly amphipathic structure (α-helical/β-sheet like) exhibited significant antimicrobial activities (Jin et al., 2005).

**FIGURE 6:**
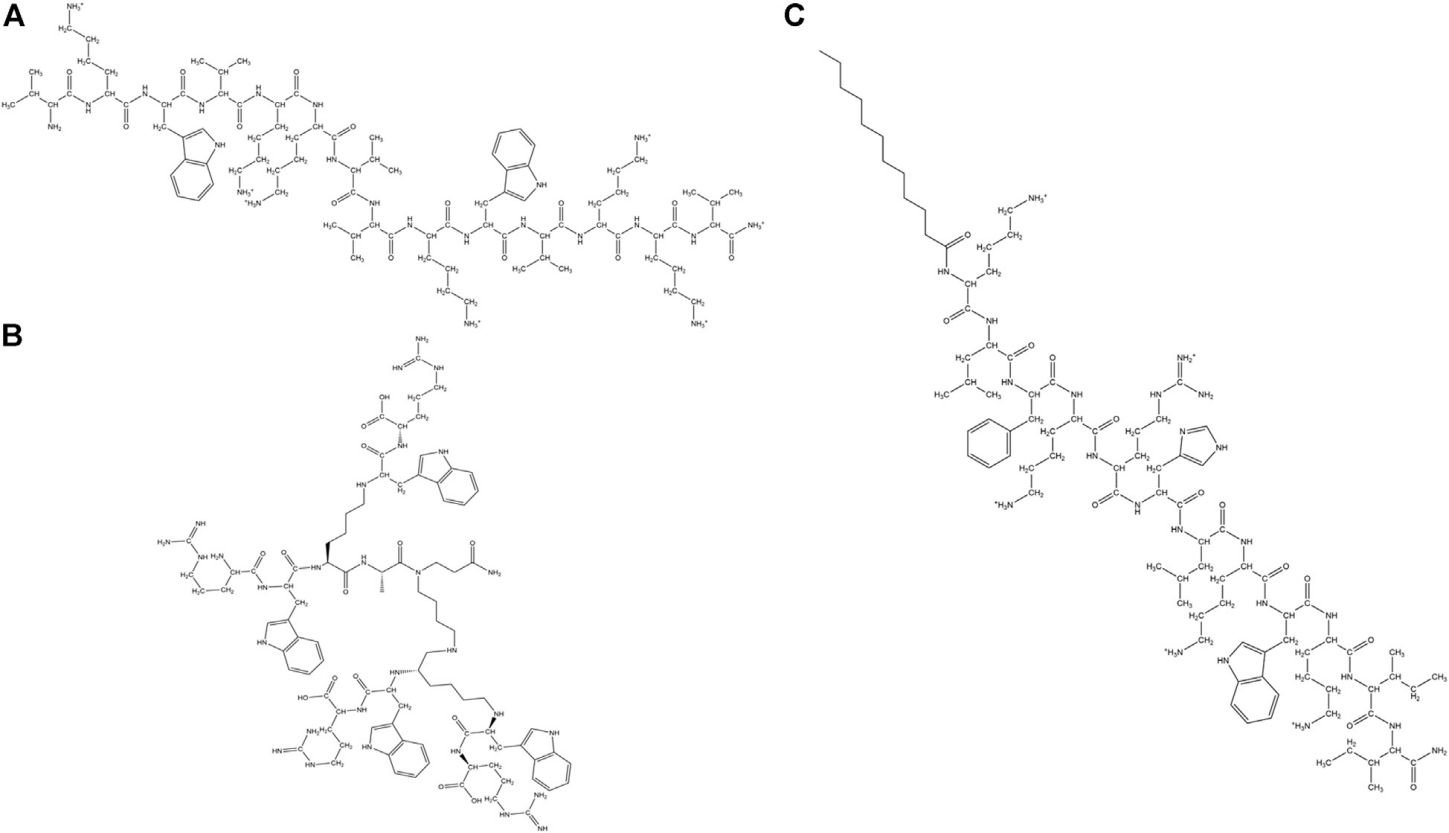
Examples of synthetic cationic AMPs. **(A)** VV-14 (Pandit et al., 2020). **(B)** Dendrimeric peptide: (RW)_4D_ (Hou et al., 2009). **(C)** C_12_-SC4 (Chu-Kung et al., 2004).

Zhang et al*.* designed α-helical cationic AMPs with the variable number of Pro residues and demonstrated that Pro had a deactivating effect on the potency of the membranolytic cationic α-helical AMPs (Zhang et al., 1999). Jadavpour et al. designed amphipathic Lys rich 7-mer, 14-mer and 21-mer peptides. 14 and 21 mer peptides exhibited high inhibition against gram-negative *E. coli* and *P. aeruginosa* and gram-positive *S. aureus*. 7-mers were inactive and unstructured, 14-mers peptides had low cytotoxicity while 21-mer peptides showed high cytotoxicity except for (KLGKKLG)_3_ and (KAAKKAA)_3_ against human erythrocytes as well as mouse fibroblasts. 14-mer and 21-mer peptides adopted helical structures as a function of the length of the peptides and the length of the hydrophobic side chain residues. The propensity of the AMPs to form helical conformation was proportional to their cytotoxicity and inversely proportional to their activity (Javadpour et al., 1996). Pandit et al*.* designed a series of short cationic heptapeptides. Peptides P4: LKWLRRL-NH_2_, P5: LRWLRRL-NH_2_ had broad-spectrum antimicrobial activity against several members of ESKAPE pathogens (*E. coli, P. aeruginosa, Klebsiella pneumoniae (K. pneumoniae*)*, S.typhi, and S.aureus*) and pathogenic fungi like *C. albicans and C.grubii*. P4 and P5 were non-hemolytic, non-cytotoxic and membranolytic in their mode of action (Pandit et al., 2018). In another study, Pandit et al. demonstrated that in a series of three 14 amino acid residue containing synthetic AMPs LL-14, VV-14 and ββ-14 having identical charge and sequence but differing in the hydrophobic amino acid residues, the length of the side-chain played an important role in the activity and the cytotoxicity of the peptides. Long hydrophobic side chain in Leu led to the best activity and highest cytotoxicity in LL-14 of all the three peptides. VV-14 was optimally active and non-cytotoxic (Pandit et al., 2021) (Figure 6A).While LL-14 and VV-14 formed α-helices in presence of membrane mimics, ββ-14 remained unstructured. In yet another study, a family of cationic, highly potent (against ESKAPE pathogens, *C. albicans, Cryptococcus grubii* (*C. grubii*)), non-cytotoxic undecapeptides were designed (Pandit et al., 2020). Huang et al. designed a series of short triblock amphiphilic peptides (K_n_F_m_K_n_) with high potency against both gram-positive (*S. aureus*) and gram-negative bacteria (*E. coli*) with low cytotoxicity towards mammalian cell lines (Huang et al., 2020).

Three cationic synthetic peptides JH8194(KRLFRRWQ), JH8195(KRLFRRLLFSMKKY) and JH8944(FKCKKVVISLRRY) were shown to have greater anticandidicidal effects than naturally occurring antifungals like Histantin 5 and Lactoferricin B (Nikawa et al., 2004). dF21-10K, the most potent AMP of the family of synthetic cationic peptides called kaxins could completely eradicate the preformed layer of biofilm formed by *C. albicans* and *Candida tropicalis* (*C. tropicalis*) (Burrows et al., 2006).

Dendrimers are tree like molecules that have radially distributed layers of branches called generations. They can form covalent or noncovalent assembly of multiple ligands and are known to manifest the dendrimeric effect or non-linear enhancement of certain properties like drug potency or bioavailability. Polylysine dendrons (PLL) are a class of dendrimers proposed by Tam et al. (Tam et al., 2002). Such molecules have an inner matrix constituted of several layers of Lys connected by the α and ε-amino groups. The amino groups of Lys that are located on the surface can bind to one or more peptides. Recently, PLL dendrons have been used as scaffolds for carrying 2 or 4 ten residue peptide (RGRKVVRRKK) which exhibited a very strong activity against *P. aeruginosa* and a very low activity against fungi (Lakshminarayanan et al., 2014). Divalent and tetravalent dendrons expressed non-linear enhancement of activity against three reference *Candida* strains and two clinical isolates of *C. albicans*. Additionally, dendrimeric AMPs retain their activity against high salt concentrations or in the presence of trypsin, blood serum and tear fluid. Small dendrimeric cationic lipopeptides acquired selective activity against *Candida sp*. and exhibited low hemolytic activities. These lipopeptides produced potassium leakage from fungal cells, caused morphological alterations of the fungal cells and inhibited activity of candidal β (1,3) glucan synthase (Janiszewska et al., 2012).

Kallenbach and coworkers designed a dendrimeric peptide (RW)_4D_ based on the short cationic peptide sequence (Figure 6B). The peptide exhibited activity against multi drug resistant strains of *S. aureus* and *E. coli* D31 (resistant against ampicillin and streptomycin) and *Actinobacter baumanni*. Additionally, the peptide was shown to inhibit *E. coli* 437 in the planktonic and biofilm states. Most bacteria in the preformed biofilm lost their viability after being treated with this peptide (Hou et al., 2009).

N terminal of the Esculatin-1b, Esc (1–18) retained the antimicrobial property of the whole native peptide (Mangoni et al., 2003b). Similarly, N-terminal 21 amino acid residue peptide from Esculatin 1a Esc(1–21)NH_2_ inhibited the growth of *Saccharomyces cerevisiae* by modulating the synthesis of proteins that are involved in the metabolism of cell membranes (Gamberi et al., 2007). The activity of Esc (1–21)NH_2_ against *P. aeruginosa* was salt tolerant. This led to its administration to *P. aeruginosa* keratitis in the murine model that caused a significant reduction of the infection (Kolar et al., 2015). Esc(1–21)NH_2_ diastereomer Esc(1–21)-1C also exhibited activity against free-living and sessile forms of *P. aeruginosa* (Cappiello et al., 2016). Biondi et al. designed Esculatin (1–21)NH_2_ analogs with the incorporation of unnatural amino acid residue α-amino isobutyric acid for improving the activity of the parent peptide towards gram-positive bacterial strains. The designed analogs improved in helicity, activity against gram-positive bacterial strains with no induction of cytotoxic effects (Biondi et al., 2017).

Gopal et al. modified a previously reported peptide HPA_3_NT_3_ (FKRLKKLFKKIWNWK) by substituting Arg and Asparagine (Asn) with Lys and two Trps with Leu to synthesize analog FKKLKKLFKKILKLK-NH_2_ with an enhanced cationic character. Both the peptides were membranolytic and considerably active against several bacterial and fungal strains. The analog was more potent against gram-positive bacterial strains and less hemolytic (4.2%) compared to the parent peptide (71%) against hRBCs (Gopal et al., 2009).

Dinh et al*.* synthesized a doubly stapled peptide Ac-DS-14W based on Alanine (Ala)/Lys model sequence Ac-KXAKAXKKXAKAXWK-NH_2_. This peptide was designed to have two oct-4-enyl staples, which cross-linked residues at positions 2 and 6 and positions 9 and 13, respectively. For comparison, two more analogs of Ac-DS-14W, one singly stapled Ac-SS-14W and the other unstapled Ac-UM-14W were synthesized. Helical content of the peptides increased with the number of staplings in the peptides. Unstapled peptide was found to be inactive against all the tested microbes. Antimicrobial activity of the peptides increased with the number of staples, though doubly stapled peptides were found to have less activity against gram-negative bacteria *K. pneumonia*. Proteolytic resistance as well as cytotoxicity of the peptides increased with the number of staples in the peptides (Dinh et al., 2015).

Castelletto et al*.* designed a surfactant-like peptide with a single Arg residue Ala_6_-Arg (A_6_R) in which both the termini were capped/uncapped. Cap-A6R had a specific activity towards gram-positive *Listeria monocytogenes* while A6R was active against all the gram-positive and negative bacterial strains. The difference in activity was attributed to the selective interaction of the peptides with the membrane mimics. (Castelletto et al., 2018).

**FIGURE 7:**
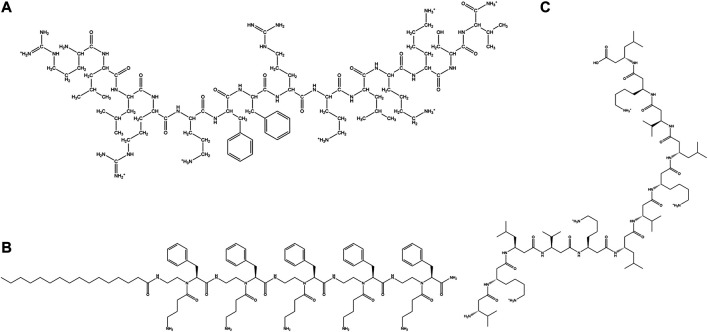
Examples of synthetic cationic AMPs containing unnatural amino acid residues. **(A)** Cbf-14–2 (Kang et al., 2017). **(B)** NB-119–2 (Hu et al., 2012). **(C)** β-amino acid containing peptide H-(β^3^-H-Val- β^3^-H-Lys- β^3^-H-Leu)_4_-OH (Liu and De Grado, 2001).

Dimeric temporin analog (TAd), all L modified temporin analog (TAL-L512) and all D temporin analog (TAD-L512) were synthesized. All the peptides showed antibacterial activity towards *S. aureus.* However, other than TAL-L512 all the other analogs were cytotoxic towards human keratinocytes (Mantyla et al., 2005). A synthetic analog of melittin named MeIP5, was shown to have very advanced pore forming ability in comparison to the parent peptide. The design involved the change of four positively charged amino acid residues in the nonpolar face of the amphipathic α-helix structure of melittin to hydrophobic amino acid residues (Wiedman et al., 2014). Reyes et al. studied the effect of trimethylation of the ε-amino groups of Lys at the selected positions of the antimicrobial hybrid peptide cecropin A-melittin KWKLFKKIGAVLKVL. Increase in Lys-(Me)_3_ residues, led to a decrease in both the antimicrobial activity against *Leishmania*, gram-positive bacteria like *S. aureus* and gram-negative bacteria like *Acinetobactor baumannii*. Membrane permeabilization and depolarization were the mechanism of action of the AMPs. The proteolytic stability of the peptides increased with the number of trimethylated Lys (Fernandez-Reyes et al., 2010). Svenson et al. designed a library of tripeptides by employing Biphenylalanine (Bip) as the central residue flanked by Arg/Arg-like amino acids. Unnatural amino acids like phenylalanine (Phe) derivatives L-2-amino-3-(4-aminophenyl)- propanoic acid (App) and L-2-amino-3-(4-guanidinophenyl) propanoic acid (Gpp) were included to establish the influence of hydrophobic bulk in direct conjunction with the cationic charge on the antibacterial property of the peptides. Tripeptides containing two Gpp residues were most active followed by those containing one Arg and another Gpp residue. All the peptides showed low haemolytic activity (Svenson et al., 2009). Hicks et al. used multiple repeats of two unnatural amino acid residues Tetrahydroisoquinolinecarboxylic acid (Tic) and Octahydroindolecarboxylic acid (Oic), spaced by two amino acid residues for constructing amphipathic α-helical AMPs. The peptides showed broad-spectrum antimicrobial activities against ESKAPE pathogens and haemolytic activities. Few of these designed AMPs showed activity in the *in vivo* mouse wound healing model (Hicks et al., 2007, 2013). Kang et al*.* designed analogs of AMP Cbf-14 by the mutation of Lys and Leu residues with unnatural amino acid analogs Ornithine (Orn) and Nor-Leucine (Nle) respectively. An all D-amino acid residue containing analog was also developed. All the peptides had similar or better activity in comparison to Cbf-14 and negligible hemolysis in sheep RBCs. Peptides adopted helical conformation in the presence of membrane mimetic environments. One of the AMPs, Cbf-14–2 (Figure 7A) exhibited *in vitro* as well as *in vivo* activity against penicillin resistant bacteria, while retaining low toxicity against mammalian cells (Kang et al., 2017).

Short lipopeptides were synthesized from tethering cationic tetrapeptide composed of one D-Phe, two L-2,4 diamino butyric acid and a L-Phe residue to fatty acids of different chain lengths. All the peptides were non-hemolytic and showed high activity towards gram-positive bacteria. (Juhaniewicz-Dębinśka et al., 2020). Chu-Kung et al. designed lauric acid derivates of three peptides AKK, SC4, and KAK. Lipidated AKK and SC4 showed improved antimicrobial potency in comparison to the non-lipidated analogs, in contrast to lipidated KAK, owing to the increased helical content of lipidated AKK with respect to KAK in the presence of microbial membrane mimetics. The cytotoxicity of lipidated AKK and KAK peptides was low in comparison to SC4 due to the ability of the later to indiscriminately adopt helical conformation in microbial and mammalian zwitterionic membrane mimic. This study concluded that lipidation could alter the antimicrobial potency and cytotoxicity of the peptides by altering the secondary structure of the peptides (Chu-Kung et al., 2004). (Figure 6C) Hu et al. synthesized several short cationic lipidated α-AA-peptides containing unnatural N-acylated-N-aminoethyl amino acid residues with broad-spectrum antimicrobial properties against clinically relevant bacterial and fungal strains. NB-123, NB-119–2 (Figure 7B), and NB-119–3 peptides exhibited better antimicrobial activity and selectivity in comparison to the clinical therapeutic pexiganan. The peptides were membranolytic in their activity which helped overcome the problem of the development of resistance against them (Hu et al., 2012). Short (RW)_3_ sequences with side chain lipidated terminal Lys residues had broad-spectrum antimicrobial activity against gram-positive and gram-negative bacteria. Both N and C-terminally lipidated peptides showed high activity against a broad range of pathogens including *P. aeruginosa* and *A. baumannii*, though the C terminal peptide had higher hemolytic activity (Albadaet al., 2012). Zhong et al. designed fatty acid derivatives of AMP Anoplin (GLLKRIKTLL-NH_2_) and its D isomer. The fatty acid chains (4–16 carbon atoms long) were added to the side chains of the D-amino acid residues. The peptides showed excellent activity against a range of bacteria, especially the multidrug resistant ones, resistance to trypsin, serum, salts, pH environments and low tendency to develop antibacterial resistance. The lipidated AMPs showed membrane activity (outer/inner membrane permeability, membrane depolarization, deformation) in addition to possessing intracellular targets. Some of the peptides possessed *in vivo* antimicrobial potency, anti-inflammatory activity and endotoxin neutralization ability (Zhong et al., 2020). Niu et al. synthesized a series of cationic lipo-γ-AA peptides with unsaturated lipid chains tethered to peptides composed of unnatural amino acid residues with positive side chains. The lipo-γ-AA peptides showed high broad-spectrum antimicrobial activities, low hemolytic activity and low resistance development abilities (Niu et al., 2012). Karlsson et al. designed cationic amphipathic 14-helical β-peptides formed from β amino acid residues with potent antifungal activity against *C. albicans*. Decamer β^3^Tyr-(Aminocyclohexane carboxylic acid (ACHC)-β^3^Valine (Val)- β^3^Lys)_3_ exhibited high potency and a moderate degree of hemolysis (Karlsson et al., 2006). Degrado and coworkers designed helical membranolytic peptides H-(β^3^-HAla-β^3^-HLys-β^3^-HVal)n-NH_2_ (where *n* = 4, 5) (Figure 7C) with high antimicrobial but low hemolytic potencies (Liu and DeGrado, 2001). AMPs containing E-vinylogous γ-amino acids in cationic (αγ)_2_ lipopeptides showed broad-spectrum antimicrobial property against several bacterial and fungal strains, when the side chains of the γ-amino acids were that of Ala, tyrosine (Tyr) and Phe. Self-assembled nanostructures played an important role in the activity and the hemolytic activity of the peptides (Shankar et al., 2013).

### Synthetic Cyclic Peptides:

**FIGURE 8:**
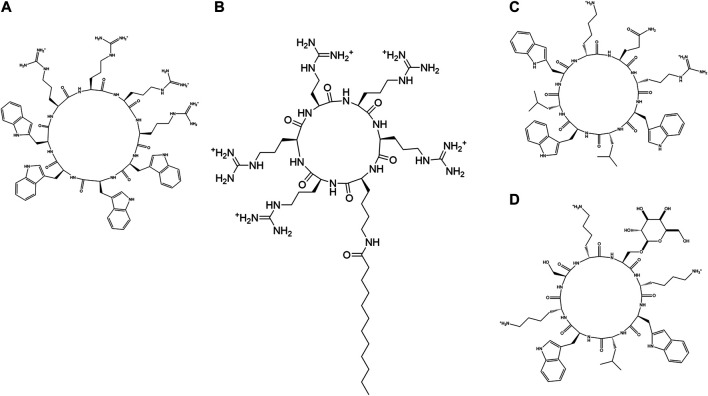
Examples of synthetic cyclic AMPs. **(A)** (R_4_W_4_) (Oh et al., 2014). **(B)** C12-R5 (Oh et al., 2014). **(C)** (kQrWlWlW) (Fernandez-Lopez et al., 2001). **(D)** (WlWkUkSk), *U* = Ser(α-Man) (Motiei et al., 2009).

Cyclic peptides are found to have a superior affinity to the target receptors as compared to that of the linear form. Firstly, cyclization improves the proteolytic susceptibility (Joo, 2012) of linear AMPs. Cyclization of peptides removes the reactive C and N-termini and acts as shielding components against the proteolytic enzymes. Secondly, cyclization improves binding the affinity of the AMPs for the microbial membranes. The flexibility of the linear AMPs, lead to an entropic penalty upon membrane binding. Cyclic AMPs with rigid structures do not incur entropic penalty upon membrane binding, which leads to its higher membrane binding affinity and hence activity. Figure 8 and Table 2 depicts some representative synthetic cyclic peptides.

Synthetic cyclic peptide (Gly)_6_ccTP, an analog of the β-stranded AMP tachyplesin, showed salt tolerant activity against several microbial strains. (Gly)6ccTP contained eight mutated Gly residues instead of hydrophobic counterparts in the parent AMP and retained all the three disulfide bridges in the cyclic peptide backbone (Tam et al., 2000). Oh et al. studied two classes of amphiphilic cyclic cell-penetrating peptides (CPPs): 1) cyclic peptides containing Trp and Arg amino acid residues and 2) fatty acylated cyclic polyarginine peptides. Cyclic (R4W4) peptide had an enhanced activity in comparison to the linear R4W4 towards MRSA (Figure 8A). Antimicrobial potency of cyclo (R4W4) (2XMIC) with tetracyclin demonstrated was enhanced by 4-8 folds against MRSA and 2-8 folds against *E. Coli* with respect to the antibiotic alone. Among fatty acylated cyclic peptides, dodecanoyl (Figure 8B) and hexadecanoyl KR5 and dodecanoyl KR6 exhibited the highest potency against MRSA. The antimicrobial potency of the peptides was in the same order as their cell-penetrating abilities (Oh et al., 2014). In another study, cyclic AMPs with 6–12 amino residues were synthesized keeping in mind Ghadiri’s alternate D, L sequence design (Figure 8A) (Bong et al., 2001; Fernandez-lopez et al., 2001; Dartois et al., 2005; Fletcher et al., 2007) and cyclized via a xylene double thioether bridge connecting a pair of cys residues placed at both ends of a linear sequence to promote aggregation and serum stability (Timmerman et al., 2005). RH11 had good activity against *P. aeruginosa* including MDR clinical isolates, good serum stability and moderate hemolysis. These peptides were membranolytic in action (He et al., 2017).

Nanotubes formed from cyclic peptides are another important class of antimicrobials. These nanotubes are stable against proteolytic degradation and easily formed on the charged membranes of bacteria. Unique supramolecular arrangements, made development of bacterial resistance against them. Ghadhiri et al. were the first to assemble cyclic peptide nanotubes by acidification of a pH-responsive octapeptide (Ghadiri et al., 1993). Cyclic peptide assemblies from cyclic gamma or chiral β^3^ amino acid residues were much more stable owing to rigid backbone of the cyclic γ amino acid residues in comparison to the α amino acid residues. Antimicrobial activity of the CNPT against microbes is based on several factors like ring size, nature of constituent amino acid residues, nature and number of positively charged amino acid residues (Chapman et al., 2012). Fernandez-Lopez et al*.* demonstrated that the antimicrobial activity of the cyclic peptides was way enhanced than their linear counterparts. Cyclic 8-mer AMPs showed better activity than the 6-mers. Antimicrobial and haemolytic activity remained the same on changing the chirality of the basic amino acids (Fernandez-Lopez et al., 2001) (Figure 8C). Motiei et al. studied membrane activity as well as *in vitro* activity of cationic cyclic D, L-α-glycopeptides bearing D-glucosamine (GlcNH_2_), D-galactose (Gal), or D-mannose (Man) glycosyl side chains. A membrane active amphiphilic parent peptide cyclic D, L-α-(WLWKSKSK) was modified to glycopeptides of the same charge but containing glycosyl side chains (Figure 8D). Glycosylation improved the activity against gram-positive bacterial strains including MSRA. The glycosylated peptides were protease resistant, had membrane permeation mode of action and cytotoxicity decided by the position and type of glycosyl residues (Motiei et al., 2009).

### Tryptophan, Proline, Histidine, Glycine Rich Synthetic Antimicrobial Peptides

**FIGURE 9:**
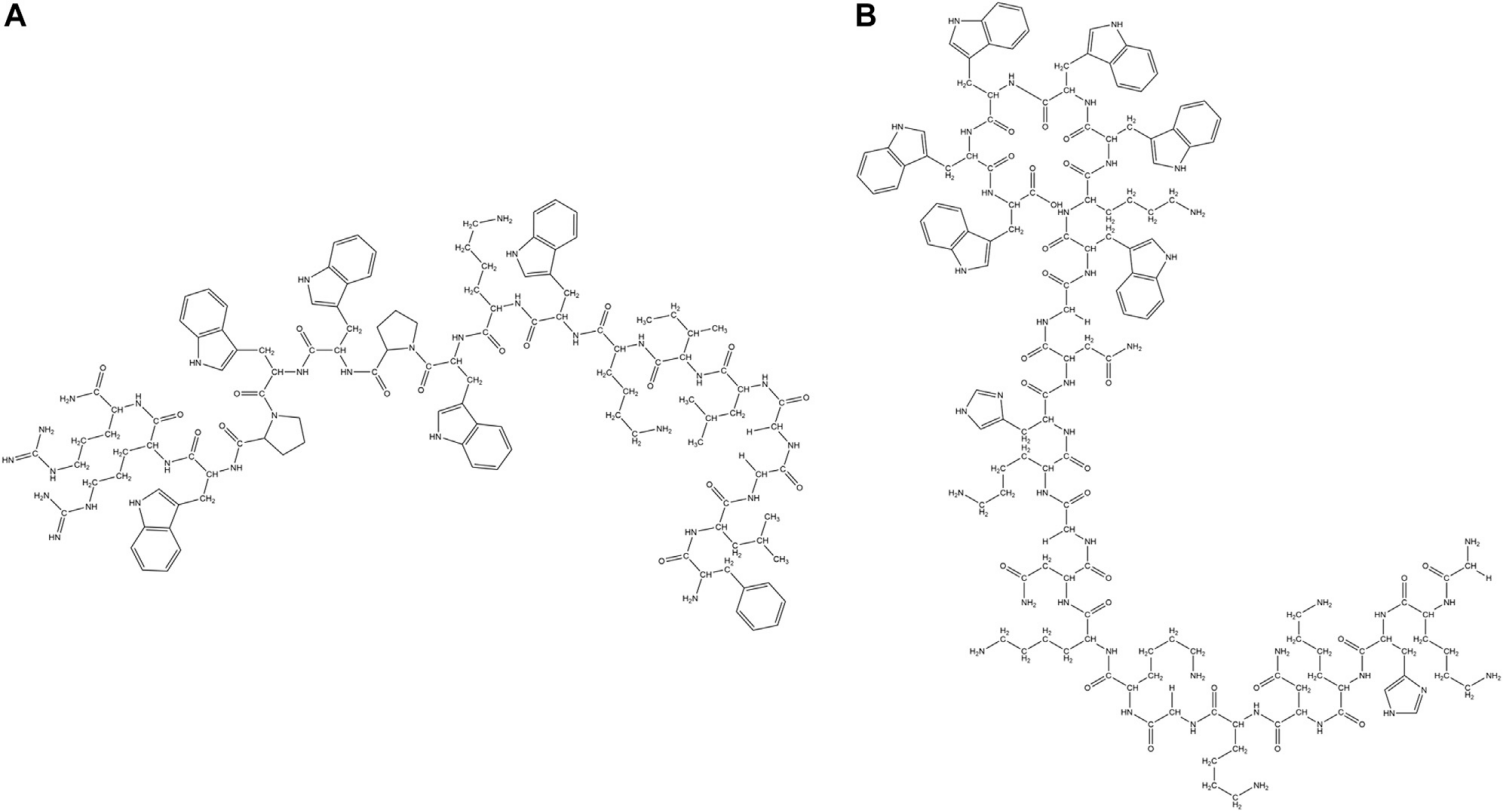
Examples of synthetic AMPs rich in specific amino acid residues (Trp, Pro, His and Gly). **(A)** Trp-Pro rich peptide: RN7-IN10 (Jindal et al., 2017). **(B)** Trp-Gly rich peptide: GKH17WWWWW (Pasupuleti et al., 2009).

Learning from nature, several synthetic peptides have been developed which are rich in certain amino acid residues like Trp, Pro, His and Gly (Figure 9; Table 2). Due to its unique characteristics, several Trp rich short peptide antibiotics have been designed. A minimum of two Trp residues with a C terminal amidation was essential for the antimicrobial property. Additionally, about six amino acid residues were required for attaining maximum antimicrobial activity against gram-negative bacteria like *E. coli* or gram-positive bacteria like *S. aureus*. Increasing the number of Trp residues beyond six, marginally increased the activity while decreasing the number of Trp residues in hexa/heptapetides by mutation with Tyr residue led to significant inactivation of the peptide, especially against gram-negative bacterial strains.

PAF26 (Acetyl-RKKWFW-NH_2_), a Trp rich antifungal hexapeptide was obtained from combinatorial chemistry, that is active against *Penicillium italicum, Penicillium digitatum* and *Botrytis cinerea* (Muñoz et al., 2006; Lopez-García et al., 2000). Cyclic analog of the PAF26 was found to be active against gram-positive bacteria like *S. aureus* and *S. sanguis* and gram-negative bacteria like *E. coli*. It is also found to be active against pathogenic fungi like *C. albicans* (Blondelle et al., 1996). LfcinB_4-9_, RRWQWR was identified as the active antibacterial component of the LfcinB (Tomita et al., 1994). Position of the Trp residues was as/more important than the number of Trp residues. Synthetic peptides KCM11(TWWRWW-NH_2_), KCM12(KWRWIW-NH_2_), KCM21(KWWWRW-NH_2_) and KCM22(WRWFIH-NH_2_) were identified from the positional screening of the synthetic peptide library and were shown to have activity against phytopathogenic bacteria *Pectobacterium* spp., *Xanthomonas* spp., and *Pseudomonas* spp. (Choi and Moon, 2009). LysH and Lys C were two cationic Trp rich peptides containing 7 and 15 amino acid residues respectively which wer**e** designed from the C terminal end of the human and egg while lysozyme. Both the peptides showed broad-spectrum antibacterial activity against gram-positive bacterial strains like *B. subtilis*, *S. aureus*, *Streptococcus zooepidemicus* and several gram-negative bacterial strains like *E. coli*, *K. pneumonia*, *P. aeruginosa*, *Serratia marcescens* (Pellegrini et al., 1997). A rationally designed synthetic hexapeptide (RW)_3_ was highly active against bacterial strains like *E. coli* and *S. aureus* (Liu et al., 2007) and pathogenic fungal strains like *Fusarium solani* and *Fusarium oxysporum* (*F. oxysporum*) (Gopal et al., 2012). Grain softness protein (MPLSWFFPRTWGKR-NH_2_) derived from the wheat grain (Phillips et al., 2011) exhibited antibacterial (*E. coli, S. aureus*) and antifungal activities (*Colletotrichum graminicola*, *R. solani*). GAK4AL, exhibited antibiotic activities against gram-negative bacterial strains like *E. coli*, *K. pneumonia*, *Shigella dysenteriae*, *Salmonella typhimurium* (*S. typhimurium*) and gram-positive bacterial strains like *S. aureus*, *Staphylococcus epidermidis* (*S. epidermidis*), *P. aeruginosa*, *B. subtilis*, *Micrococcus luteus* (*M. luteus*) (Won et al., 2011)*.* PW2, a synthetic peptide isolated from the phage display library (HPLKQYWWRPSI) exhibited antifungal activity against *Eimeriatenella*, *Eimeria acervulina*, *C. albicans*, *A. nidulans* (*Aspergillus nidulans*) (Da silva et al., 2002).A synthetic linear and cyclic analog of the Indolicidin peptide CP-11(ILKKWPWWPWRRK-NH_2_) and cycloCP-11, with an increased positive charge and amphipathicity was shown to have enhanced activity against gram-negative bacteria. Both the peptides were found to have similar kind of structure and binding to membrane mimics, thus resulting in similar activity. However, the cyclic analog had much enhanced resistance to protease degradation in comparison to the linear analog (Rozek et al., 2003). The carboxymethylated derivative CP-11C exhibited improved activity against gram-negative bacteria and *C. albicans*, while it retained activity against *Staphylococci* in comparison to Indolicidin (Falla and Hancock, 1997). Hybrid peptides RN7-IN10 (Figure 9A), RN7-IN9, RN7- IN8, and RN7-IN6, based on two natural AMPs Indolicidin and Ranalexin, were shown to have broad-spectrum antimicrobial activity against *S. aureus*, MRSA, and *E. coli*. The hybrid AMPs were non-cytotoxic and non-hemolytic, and exhibited potent antibacterial activity against 30 different pneumococcal clinical isolates. Hybrid AMPs were more potent against *K. Pneumoniae* than standard drugs like erythromycin and ceftriaxone (Jindal et al., 2015). A truncated 13 residue synthetic fragment (Puro A), containing the Trp rich domain of Puroindoline A was shown to exhibit native-like antimicrobial property and hence considered to be responsible for the bactericidal activity of Puroindoline A (Jing et al., 2003). Pasupuleti et al. designed two peptides GKHKNKGKKNGKHNGWK and KNKGKKNGKH from kininogen and added various length of Trp and Phe tags (Figure 9B). The antimicrobial potency of the AMPs increased with the length of the tags, which was related to the efficacy of lysis of the bacterial walls and to more pronounced bacterial lipopolysaccharide binding. The end tagged analogs were protease, salt, serum, and anionic macromolecular scavenger tolerant (Pasupuleti et al., 2009). Lee et al*.* designed a series of peptides based on the basic sequence (XWZX)_n_ (*n* = 2–4, X: Lys or Arg, Z: Leu, Tyr, Val, or Gly). All the peptides showed low cytotoxicity and high antimicrobial properties against drug-susceptible and drug-resistant strains through membranolytic mechanisms (Lee et al., 2018). N terminal modified metallocenoyl {ferrocenoyl [FcC(O)-] or ruthenocenoyl [RcC(O)-]} derivatives of all L and diastereomeric WRWRW-NH_2_ were synthesized. Mostly all the derivatives were active against gram-positive bacterial strains like *S. aureus*, MRSA and *B. subtilis*. Most of the peptides were inactive against gram-negative strains and had limited hemolytic activity. Diasteromeric peptides had better activity than the all L peptides while FcC(O)- derivatives were more potent than the corresponding RcC(O)- derivatives (Albada et al., 2014). Four ultrashort Arg and Trp based peptides RW, WR, WRW and RWR were synthesized and covalently bound to two different trivalent scaffold molecules namely 1,3,5-tris(azidomethyl)- benzene and a 1,3,5-tris(azidomethyl)-2,4,6-triethylbenzene using the copper(I)-catalyzed alkyne–azide cycloaddition (CuAAC) reaction (Figure 9C **)** to check the effect of preorganization of the AMPs on their antibacterial property. The AMPs were more active against the gram-positive compared to the gram-negative bacteria. Comparison of the activity with the Linear (RW)_3_-NH_2_ suggests that the presence of certain number of amino acids in the close vicinity is more important than their relative spatial orientation (Hoffknecht et al., 2015). High antimicrobial property was obtained by increasing the hydrophobic volume of the side chains of the amino acid residues. Hydrophobic chains such as Bip and 2.5.7 tri-tert-butyl Trp (Tbt) generated the most potent peptides when associated with either C terminal benzyl esters or amides (Hang et al., 2004). These peptides had much higher selectivity against gram-positive bacteria in comparison to the gram-negative bacteria. Applying Tbt amino acid residue instead of Trp in the RXR motif, highly active AMPs were obtained. An AMP RWRWRWA-(Bpa), with a photoactive group p-benzoyl-phenylalanine (Bpa) at the C- terminus was attached to a reverse osmosis (RO) membrane surface, upon UV radiation. Bacterial viability on the modified surfaces decreased by 55% with reduction of biofilm caused by *P. aeruginosa*. The viability of gram-positive bacteria *E. faecalis* on the peptide coated surface exhibited 29–40% bacterial inhibition (Mao et al., 2018). Hybrid Trp rich cationic peptides KU2 and KU3, generated from the fusion of two peptides KABT-AMP and Uperin 3.6 showed high antifungal potency against *C. albicans*. Though the peptides were non-hemolytic, they showed significant toxicity against human epithelial cell lines (Lum et al., 2015).

Synthetic Pr-AMP PR-26, derived from the N terminus of the PR-39 exhibited higher activity towards enteric gram-negative bacteria. Pr-26 potentiated neutrophil phagocytosis of *Salmonella choleraesuis* and decreased the invasion of *S. typhimurium* into the intestinal epithelial cells. Pr-26 facilitated the pore forming AMPs like magainin A, thus potentiating the innate host defense against the gram-negative bacterial infections (Shi et al., 1996). Synthetic analogs of Pr-AMP Bac5, namely Bac(1–25) and Bac(1–31) exhibited excellent activity towards *E. coli*, while Bac(1–15) was inactive. Bac(1–25) and Bac(1–31) were shown to prevent protein synthesis *in vitro* and *in vivo*. PMAP-23 amphipathic α-helical peptide isolated from porcine was modified by incorporation of Trp residues at positions 10, 13 and 14 of the hydrophobic face of the peptide to generate improved antifungal activities. This increased the fungicidal and antitumor activities of the peptide (Lee et al., 2002).

The His and Gly rich synthetic homodimer di-K19Hc designed from domain 1 of AMP Halocidin, was salt-tolerant and active against bacteria, yeast and filamentous fungi, such as *C. albicans, C. neoformans* (*Cryotococcus neoformans*)*, A. niger* (*Aspergillus niger*)*, A. terreus* (*Aspergillus terreus*) *and F. oxysporum* (Jang et al., 2006). One reported method to increase the salt tolerance of the synthetic AMPs was to replace the amino acid residues like His and Trp with bulky residues like β-naphthylalanine and β-(4,40 -Bip (Yu et al., 2011). However, these substitutions induced cytotoxicity and hemolytic activities in the peptides. Pep6 (FRLKFH), obtained from combinatorial chemistry, is a hexapeptide rich in Phe and His residues that is active against several plant pathogens like F*. oxysporum f. sp. lycopersici, Rhizoctonia solani, Ceratocystis fagacearum and Pythium ultimum* (Reed et al., 1997).

N terminal tethering of fatty acids (palmitic/undecanoic) to the inactive diastereomers of Gly rich magainin (D4-magainin) or to very weakly active diastereomeric lytic peptide D-K5L7 conferred antifungal activity against *Cryptococcus neoformans*. Particularly D-K_5_L_7_-UA showed potency against all the different microbes like bacteria, fungi and yeast (Avrahami and Shai, 2003). AMP derivatives were synthesized by simultaneous introduction of positive charges as well as Pro residues in a typical antimicrobial peptide F5W-magainin 2 (MG2). The former and the later contributed to the increase in the antimicrobial activity and the reduction in the cytotoxicity of the peptides respectively, increasing the overall therapeutic index of the peptides. Results were sensitive to the position of the Pro substitution. The antimicrobial mechanism involved Lipopolysaccharide binding, membrane permeabilization, and DNA (Azuma et al., 2020).

## AMP Delivery Vehicles

Another major concern in the commercialization of the AMPs as therapeutics arises from their modes of administration/delivery. It is now an accepted fact that the efficacy of any drug depends on its mode of delivery. In these lines, nanostructure based drug delivery systems have been found to be particularly promising. The nanostructured AMPs are more potent as they enhance the bio-availability of the drug, facilitate sustained release and improve retention of the AMPs in the bloodstream due to their enhanced retention and permeability effect. Poorer renal clearance of nanostructured AMPs leads to full exploitation of the materials in the blood stream due to enhanced retention and permeability effect (EPR effect). Nanoscale sized delivery vehicles improve the half-life and the stability of the AMPs. Table 3 summarizes some nanostructured AMPs.

**TABLE 3:** Antimicrobial peptides: delivery vehicles, peptide hydrogels and peptide-nanoparticle conjugates.

Name of the peptides	Sequence of the peptides	Physiochemical properties	Mechanism of action	Target microbes	References
-	FF	2 aa res	Membrane permeation and depolarization	*E. coli*, *R. radiobacter*, *S. epidermidis*, *L. monocytogenes*	Schnaider et al. (2017)
Fmoc-peptides	FmocFF, FmocFFKK, FmocFFFKK, FmocFFOO	-	-	*S. aureus*, *S. Epidermidi*, *E. coli, P. aeruginosa*	McCloskey et al. (2017)
C16-P3R	C16-PRRR-NH2	4 aa res; +3 net charge; Arg rich (75%)	-	*E. coli*, *P. aeruginosa*, *S. aureus*, *S. epidermidis*, *C. albicans*	Sikorska et al. (2018)
Double chain lipopeptide	(C10)2Dap-KKK-NH2 Dap—Diaminopimelic acid	4 aa res; +3 net charge; Lys rich (75%)	-	*S. aureus*, *S. epidermidis*, *E. coli*, *P. aeruginosa*, *C. albicans*	Stachurski et al. (2019)
MAX1	VKVKVKVKVDPPTKVKVKVKV-NH2	20 aa res; +9 net charge; Val rich (40%), Lys rich (45%)	-	*E. coli*, *K. pneumonia*, *S. aureus*, *S. epidermidis*, *S. pyogenes*	Salick et al. (2007)
MARG1	VKVKVRVKVDPPTKVKVRVKV-NH2	20 aa res; +9 net charge; Val rich (45%), Lys rich (30%)	-	*Methicillin-resistant S. aureus*	Salick et al., (2009)
PEP6R	VKVRVRVRVDPPTRVRVRVKV-NH2	20 aa res; +9 net charge; Val rich (45%), Arg rich (30%)	Membranolytic	*E. coli*, *S. aureus*	Veiga et al. (2012)
CASP-K6	K6(SL)6K6	24 aa res; +12 net charge; Lys rich (50%), Ser rich (25%), Leu rich (25%)	Membrane disruption	*P. aeruginosa*	Sarkar et al. (2019)
WK_3_ (QL)_6_K_2_	CH3CO-WKKKQLQLQLQLQLQLKK-CONH2	18 aa res; +5 net charge; Leu rich (33%), Gln rich (33%), Lys rich (28%)	-	*S. aureus*	Jiang et al. (2015)
P5	H_2_N-(CH_2_)_5_-CONH-Phe-CONHC_12_	-	-	*S. aureus*, *E. coli*	Nandi et al. (2017)
P1	Boc-AUDA-Phe-COOH, (AUDA = 11-aminoundecanoic Acid)	-	-	*E. coli*, *P. aeruginosa*, *S. aureus*	Baral et al. (2016)
PAF26	Ac-RKKWFW-NH2	6 aa res; +3 net charge; Trp rich (33%), Lys rich (33%)	Interaction of the peptide hydrogel with microbial cell wall	*C. albicans*, *S. aureus*, *E. coli*	Cao et al. (2019)
CG_3_R_6_TAT nanoparticles	Cholesteryl chloroformate grafted onto the N-terminus of the G_3_R_6_TAT (GGGRRRRRRYGRKKRRQRRR), TAT: YGRKKRRQRRR	-	Inhibition of cell wall synthesis; damage to the cell wall; membrane destabilization	*S. aureus, methicillin-resistant S. aureus*, *B. subtilis*, *E. faecalis*, *vancomycin-resistant E. faecalis*, *S. haemolyticus*, *C. tropicalis*, *C. albicans*	Liu et al. (2009)
G_3_R_6_-TAT functionalized colloid siver-nanoparticles (Ag-pep)	G_3_R_6_TAT (GGGRRRRRRYGRKKRRQRRR), TAT: YGRKKRRQRRR funtionalized with silver nanoparticles	-	-	*E. coli*, *B. subtilis*, *C. albicans*	Liu et al. (2013)
AgNP-OA1 conjugate	Silver nanoparticles functionalized with OA1 (VVKCSYRLGSPDSQCN)	-	Predominantly secondary mechanisms such as interactions with the DNA, followed by other mechanisms that arrest the transcription and translation pathways	*E. coli*	Pal et al. (2016)
Cecropin Melittin (CM)-NPs	Fe_3_O_4_ (SPION)@gold core-shell nanoparticles functionalized with Cecropin Melittin (CM) (KWKLFKKIGAVLKVLC)	Partially membranolytic	-	*E. coli*, *S. aureus*	Maleki et al. (2016)
PA-1-AgNPs	Silver nanoparticles functionalized with peptide PA-1 [Ac-NH-E (NH-CH_2_-CHO)-EEEAAAVVVK(C_16_)-CONH_2_]	-	-	*E. coli*	Pazos et al. (2016)
Peptide-AuNPs-alginate bio-hydrogel	AuNP-alginate solution functionalized with peptide solution Nisin (ITSISLCTPGCKTGALMGCNMKTATCHCSIHVSK)	-	-	*S. aureus*, *B. cereus*, *E. faecalis*	Otari et al. (2016)

**FIGURE 10:**
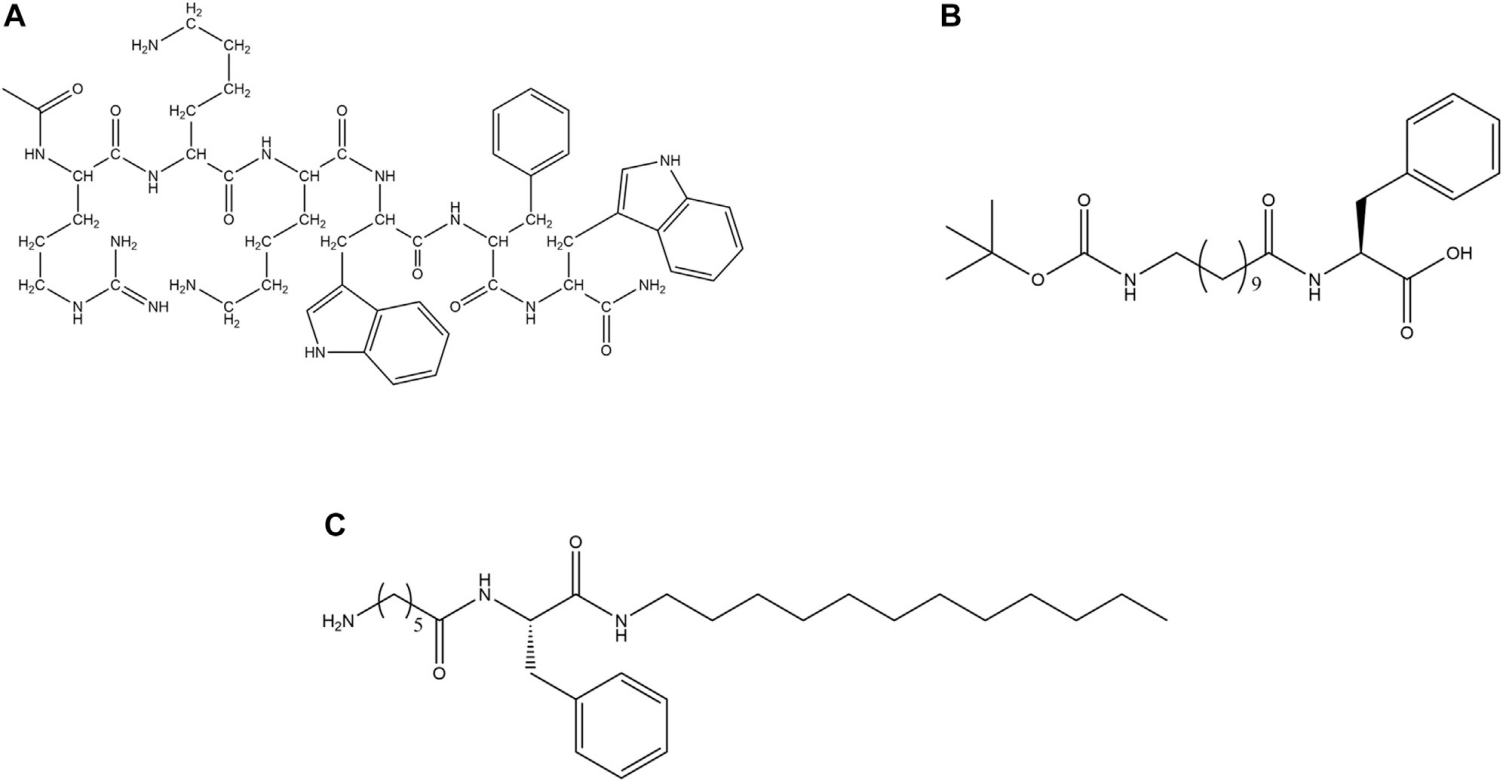
AMPs forming hydrogels. **(A)** PAF26 (Cao et al., 2019). **(B)** Boc-AUDA-Phe-COOH (P1), (AUDA = 11-aminoundecanoic acid) (Baral et al., 2016). **(C)** H_2_N-(CH_2_)_5_-CONH-Phe-CONH-C_12_ (P5) (Nandi et al., 2017).

Certain small AMPs have the ability to self-assemble and form hydrogel (Figure 10). In the literature, there are reports of ultrashort AMPs (McCloskey et al., 2017; Schnaider et al., 2017), AMPs containing lipid chains (Sikorska et al., 2018; Stachurski et al., 2019) and non-natural amino acids (Melchionna et al., 2016) which have the ability to form macroscopic hydrogels. Hydrogels impregnated with the AMPs act as a protective shield and prevent the enzymatic, thermal and chemical degradation of the AMPs (Moorcroft et al., 2020). These hydrogels may be constituted by synthetic polymers (Osada and Kajiwara, 2001), natural polymers like collagen, silk, or peptides (Jonker et al., 2012).

MAXI, a twenty residue peptide, having two eight residue strands of alternating Val and Lys amino acids connected by a tetrapeptide sequence (-VDPPT-), could form a hydrogel composed of a network of interconnected fibrils rich in β-sheet, in DEMN cell culture at pH 7.4. The fibrils were composed of bilayers of intermolecularly H-bonded hairpins. This rigid hydrogel with β-hairpin conformation could kill both gram-positive and gram-negative bacteria through membranolytic mechanism of action (Salick et al., 2007) In a separate study, Salick et al. designed a 20 residue amphiphilic β-hairpin peptide MARGI, which self-assembled into a fibrillar network resulting in a rigid hydrogel. This peptide contained two Arg groups at the 6th and 17th positions. The enhanced activity of MARGI over MAX1 against MRSA suggested that the inclusion of Arg might be critical for the enhanced action (Salick et al., 2009). Veiga et al. reported an optimized peptide containing six Arg residues, PEP6R from PEP8R, which folded into an amphiphilic β-hairpin conformation in buffer solution, rapidly self-assembling into a β-sheet rich fibrillar network, forming a hydrogel. The hydrogel exhibited distinct antibacterial efficiency toward *E. coli* and *S. aureus* via membranolytic mechanism. Also, the hydrogel was found to have a shear-thinning property and, thus could be used as a potential injectable material (Veiga et al., 2012).

CASP-K6, a cationic self-assembling AMP formed nano-fibrous hydrogel with membrane disrupting antimicrobial activity against *P. aeruginosa* colonies. CASP-K6 was a proteolytically stable and injectable hydrogel (Sarkar et al., 2019). Jiang et al. demonstrated that the antimicrobial activity of the hydrogels was related to their surface and rheological properties (Jiang et al., 2015).

AMP PAF26 formed hydrogel (Figure 10A) that exhibited antimicrobial activity against *C. albicans, S. aureus, and E. coli* through a membrane disruptive mechanism of action (Cao et al., 2019). Co-assembled hydrogels obtained from Fmoc-Phe and Fmoc -Leu amino acid derivatives showed activity against gram-positive bacteria through membrane disruptive process. Baral et al. reported a proteolytically stable N-Boc protected dipeptide (P1: Boc-AUDA-Phe-COOH) (Figure 10B) based injectable hydrogel, which showed excellent antibacterial activity against gram-negative bacteria (*E. coli and P. aeruginosa*), and biocompatibility with human red blood cells and human fibroblast cells (Baral et al., 2016). A series of five different peptide amphiphiles with a long fatty acyl chain of general chemical formula [H_2_N-(CH_2_)_n_CONH-Phe-CONHC_12_ (n = 1-5, C_12_ = dodecylamine)] (Figure 10C **)** formed hydrogels at pH 6.6. Hydrogels with longer alkyl chain lengths were found to have higher membrane affinity and antibacterial activity against both gram-positive (*B. subtilis and S. aureus*) and gram-negative bacteria (*E. coli*). These hydrogels induced a small amount of hemolysis at their MIC for *B. subtilis* and *E. coli* and exhibited proteolytic resistance to enzymes proteinase K and chymotrypsin (Nandi et al., 2017).

**FIGURE 11:**
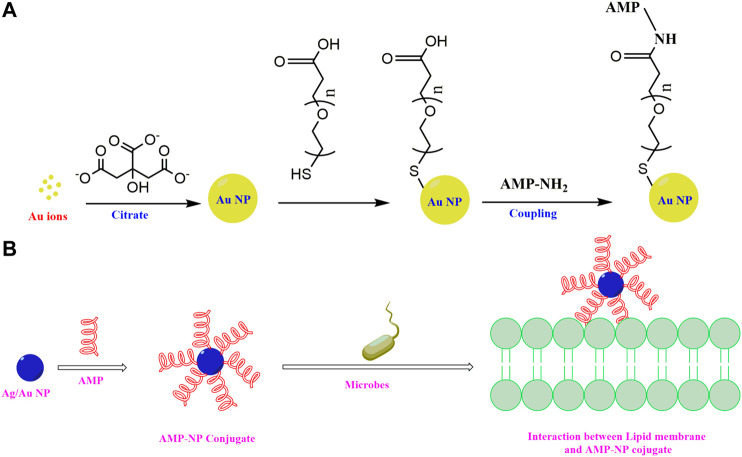
Schematic of **(A)** Stepwise synthesis of AMP-AuNP conjugates and **(B)** Formation of AMP-NP conjugates and their action on lipid membrane of the microbes.

Antimicrobial efficacy of AMP-NP systems depends on the rate, extent and the method of controlled release of the antimicrobials. Gold nanoparticle (Au-NP) based micro/nano structures are one of the most commonly used drug delivery systems owing to their biocompatibility with the mammalian systems due to their chemical inertness and stability (Rajchakit and Sarojoni, 2017). Gold nanoclusters (Au-NC) retain the biocompatibility of the AU-NP with an additional luminescent property that helps in bioimaging purposes. Antimicrobial activity of Au-NCs occur due to membrane disruption, DNA damage and generation of intracellular reactive oxygen species. Silver, oxides of metals such as titanium, copper, zinc and iron, are some of the excellent antimicrobial agents known (Singh et al., 2008; Raghunath and Perumal, 2017). However, despite their excellent antimicrobial properties, many nanoparticle based antimicrobial agents have potential cytotoxicity that needs to be addressed. Immobilization of AMPs to metallic nanoparticles reduces cytotoxicity and improves the antimicrobial potency of the individual components synergistically (Allahverdiyev et al., 2011). Some of the inherent drawbacks of AMPs, such as susceptibility to proteases and poor permeability across biological barriers, are also eliminated on conjugation with nanoparticles (Rajchakit and Sarojini, 2017). Figure 11 schematically represents the synthesis of AMP-NP conjugates.

Cell-penetrating peptide G_3_R_6_TAT (Liu et al., 2009) was used as the stabilizer and reductant to produce colloid silver nanoparticles. Though the antimicrobial activity of the Ag-peptide was lower (against *E. Coli, B. subtilis* and *C. albicans*) compared to Ag-Citrate, Ag-SDS and Au@Ag, the Ag-peptide was much less hemolytic compared to the others (Liu et al., 2013). Pal et al*.* conjugated cationic antimicrobial peptide Odorranain-A-OA1 (VVKCSYRLGSPDSQCN) extracted from the skin of Chinese odorous frogs with AgNPs. AgNP-OA1 was more potent against *E. coli* and less cytotoxic in comparison to the AgNP alone (Pal et al., 2016). Maleki et al*.* reported the preparation of biocompatible antimicrobial SPION@gold core shell NPs based on the covalent immobilization of the AMP cecropin-melittin (CM). Antimicrobial activities of the AMP-NP conjugate against gram-positive (*S. aureus*) and gram-negative (*E. Coli*) bacteria were much better than the soluble AMPs. AMP-NPs demonstrated better destruction of the microbial membranes and less hemolytic activity compared to bare NPs (Maleki et al., 2016). Pazos et al. designed PA nanofibers modified with functional groups that induced the formation of AgNPs over PA fibres. Peptide PA1 had an N-terminal aldehyde while the control peptide PA2 did not. Both the peptides formed nanofibres and on treating with Tollens reagent formed PA-AgNPs. Metallized PA nanofibers inhibited the growth of *E. Coli* inoculated in LB medium, whereas PA nanofibers did not. PA nanofibers were 30 times less toxic to the eukaryotic cells compared to the bacterial cells. Additionally, PA1-AgNPs formed antimicrobial gels with CaCl_2_ (Pazos et al., 2016). Otari et al. designed gold-nanoparticle alginate (Au-peptide-alginate) biohydrogel using a thermostable AMP nisin, effective against a variety of gram-positive bacteria. Au-peptide-alginate biohydrogel had significant antimicrobial activity against human bacterial pathogens *S. aureus*, *B. cereus* (*Bacillus cereus*), and *E. faecalis*, while the AuNP alginate did not (Otari et al., 2016).

## Conclusion

With the development of rapid resistance in microbes against standard antibiotics, the ability of the AMPs to invoke delayed resistance makes them an extremely potential therapeutic agent. Several AMPs in combination with conventional antibiotics have been shown to inhibit multiple drug-resistant strains which were resistant to the antibiotic alone, due to synergistic effect. Synthetic AMPs have been shown to have improved potency, diminished cytotoxicity, increased protease-resistance over the natural AMPs. A lot of potential therapeutic molecules can be developed by taking inspiration from the vast repertoire of AMPs that nature has to offer. Understanding the mechanisms of action of the AMPs will enable in the design of synthetic AMPs with improved specificity against specific microbial strains as well.

## Glossary

Ala: Alanine
ACHC: Aminocyclohexanecarboxylic acid
AMPs: Antimicrobial peptides
App: L-2-amino-3-(4-aminophenyl)-propanoic acid
Arg: Arginine
Bip: Biphenylalanine
Asn: Asparagine
CCK: Cyclic cysteine knot
CLPs: Cyclic lipopeptides
CPPs: Cell penetrating peptides
Cys: Cysteine
Gly: Glycine
Gpp: L-2-amino-3-(4-guanidinophenyl) propanoic acid
His: Histidine
Leu: Leucine
Lys: Lysine
MDR: Multi drug resistant
Nle: Nor-Leucine
NRPs: Non-ribosomal protein synthetases
Oic: Octahydroindolecarboxylic acid
Orn: Ornithine
PINs: Puroindolins
Phe: Phenylalanine
PLL: Polylysine dendrons
Pr-AMPs: Proline rich AMPs
Pro: Proline
ROS: Reactive oxygen species
Tbt: tri-tert-butyl tryptophan
Tic: Tetrahydroisoquinolinecarboxylic acid
Thr: Threonine
TrAMPs: Tryptophan rich AMPs
Tyr: Tyrosine
Trp: Tryptophan
Val: Valine
*A. nidulans*: *Aspergillus nidulans*
*A. niger*: *Aspergillus niger*
*A. terreus*: *Aspergillus terreus*
*B. cereus*: *Bacillus cereus*
*B. subtilis*: *Bacillus subtilis*
*C. albicans*: *Candida albicans*
*C. neoformans*: *Cryptococcus neoformans*
*C. graminicola*: *Colletotrichum graminicola*
*C. grubii*: *Cryptococcus neoformans var. grubii*
*C. tropicalis*: *Candida tropicalis*
*E. coli*: *Escherichia coli*
*E. faecalis*: *Enterococcus faecalis*
*F. oxysporum*: *Fusarium oxysporum*
*K. pneumoniae*: *Klebsiella pneumoniae*
*M. luteus*: *Micrococcus luteus*
*MRSA*: *Methicillin resistant Staphylococcus Aureus*
*MSSA*: *Methicillin sensetive Staphylococcus Aureus*
*P. aeruginosa*: *Pseudomonas aeruginosa*
*P. oryzae*: *Pleomorphomonas oryzae*
*R. solani*: *Rhizoctonia solani*
*S. aureus*: *Staphylococcus aureus*
*S. dysenteriae*: *Shigella dysenteriae*
*S. epidermidis*: *Staphylococcus epidermidis*
*S. typhimurium*: *Salmonella typhimurium*
*S. typhi*: *Salmonella typhi*
